# Key Aging-Associated Alterations in Primary Microglia Response to Beta-Amyloid Stimulation

**DOI:** 10.3389/fnagi.2017.00277

**Published:** 2017-08-31

**Authors:** Cláudia Caldeira, Carolina Cunha, Ana R. Vaz, Ana S. Falcão, Andreia Barateiro, Elsa Seixas, Adelaide Fernandes, Dora Brites

**Affiliations:** ^1^Neuron Glia Biology in Health and Disease, Research Institute for Medicines (iMed.ULisboa), Faculty of Pharmacy, Universidade de Lisboa Lisbon, Portugal; ^2^Department of Biochemistry and Human Biology, Faculty of Pharmacy, Universidade de Lisboa Lisbon, Portugal; ^3^Obesity Laboratory, Instituto Gulbenkian de Ciência Oeiras, Portugal

**Keywords:** Alzheimer’s disease, amyloid-β peptide, neuroinflammation, aged-cultured microglia, inflammatory-microRNAs, M1/M2 microglia subtypes, CD11b, CD86

## Abstract

Alzheimer’s disease (AD) is characterized by a progressive cognitive decline and believed to be driven by the self-aggregation of amyloid-β (Aβ) peptide into oligomers and fibrils that accumulate as senile plaques. It is widely accepted that microglia-mediated inflammation is a significant contributor to disease pathogenesis; however, different microglia phenotypes were identified along AD progression and excessive Aβ production was shown to dysregulate cell function. As so, the contribution of microglia to AD pathogenesis remains to be elucidated. In this study, we wondered if isolated microglia cultured for 16 days *in vitro* (DIV) would react differentially from the 2 DIV cells upon treatment with 1000 nM Aβ_1–42_ for 24 h. No changes in cell viability were observed and morphometric alterations associated to microglia activation, such as volume increase and process shortening, were obvious in 2 DIV microglia, but less evident in 16 DIV cells. These cells showed lower phagocytic, migration and autophagic properties after Aβ treatment than the 2 DIV cultured microglia. Reduced phagocytosis may derive from increased CD33 expression, reduced triggering receptor expressed on myeloid cells 2 (TREM2) and milk fat globule-EGF factor 8 protein (MFG-E8) levels, which were mainly observed in 16 DIV cells. Activation of inflammatory mediators, such as high mobility group box 1 (HMGB1) and pro-inflammatory cytokines, as well as increased expression of Toll-like receptor 2 (TLR2), TLR4 and fractalkine/CX3C chemokine receptor 1 (CX3CR1) cell surface receptors were prominent in 2 DIV microglia, while elevation of matrix metalloproteinase 9 (MMP9) was marked in 16 DIV cells. Increased senescence-associated β-galactosidase (SA-β-gal) and upregulated miR-146a expression that were observed in 16 DIV cells showed to increase by Aβ in 2 DIV microglia. Additionally, Aβ downregulated miR-155 and miR-124, and reduced the CD11b+ subpopulation in 2 DIV microglia, while increased the number of CD86+ cells in 16 DIV microglia. Simultaneous M1 and M2 markers were found after Aβ treatment, but at lower expression in the *in vitro* aged microglia. Data show key-aging associated responses by microglia when incubated with Aβ, with a loss of reactivity from the 2 DIV to the 16 DIV cells, which course with a reduced phagocytosis, migration and lower expression of inflammatory miRNAs. These findings help to improve our understanding on the heterogeneous responses that microglia can have along the progression of AD disease and imply that therapeutic approaches may differ from early to late stages.

## Introduction

Alzheimer’s disease (AD) is the most common dementing disorder in the elderly affecting around 35.6 million people worldwide and is expected to duplicate in the years to come (Prince et al., 2013). Amyloid precursor protein (APP) expression is elevated in AD, and increased amyloidogenic cleavage has been considered to cause the deposition of extracellular β-amyloid (Aβ) plaques (Rubio-Perez and Morillas-Ruiz, 2012). Deposition of Aβ was shown to trigger the activation of both astrocytes and microglia leading to the production of pro-inflammatory cytokines, such as interleukin (IL)-1β and tumor necrosis factor (TNF)-α, among other inflammatory mediators (Wyss-Coray, 2006), thus generating neuroinflammation and contributing to AD progression and severity (Heneka et al., 2015). Nevertheless, treatment with nonsteroidal anti-inflammatory drugs (NSAIDs) has consistently failed in efficacy in AD patients (von Bernhardi, 2010), unless administered early in the disease (Varvel et al., 2009). Lately, it was shown that fenamate NSAIDs decreased microglia activation and IL-1β processing in rodent models, which led the authors to suggest that they may be repurposed as inhibitors of the NOD-like receptor family pyrin domain containing 3 (NLRP3) inflammasome (Daniels et al., 2016). Microglial malfunction has been associated with AD pathogenesis. It has been indicated to contribute to changes in cell microenvironment and to precede or facilitate AD onset (Regen et al., 2017). However, the underlying molecular mechanisms of microglia failure in AD pathogenesis remain to be identified.

Microglia are the first line and the main immune defense against disease and injury in the central nervous system (CNS). When activated by stress stimuli the cells migrate and restrict the damage by surrounding the site lesion and by clearing cellular debris by phagocytosis. Microglia are activated by oligomeric and fibrillar species of Aβ, as well as by molecules derived from degenerating neurons (Mizuno, 2012), and have been described to play a key role in removal of Aβ from the brain (Morgan, 2009). However, microglia is differently polarized depending on the stimuli and the time of exposure, reason why in some circumstances the phagocytic and inflammatory phenotypes may alternate (Silva et al., 2010). It is well recognized that microglia may comprise a family of cells with diverse phenotypes exerting beneficial or destructive effects (Schwartz et al., 2006). Other studies also propose that age-dependent neuroinflammatory changes trigger decreased neurogenesis and cognitive impairments in AD (Lynch et al., 2010; Varnum and Ikezu, 2012). In addition, it has been claimed that these cells lose their ability to phagocytose Aβ with age and disease progression, and that in late disease stages inflammation no longer exists (Floden and Combs, 2011; Wojtera et al., 2012). As so, there is an urgent need to understand age-dependent changes in microglia function and associated differences in responding to stimuli to better recognize the diverse roles that these cells may have in early and late-stages of AD. Recent evidences showed that endogenous microRNAs (miRNAs), a subset of small noncoding RNA molecules that play an important role in the regulation of gene expression at the posttranscriptional level, are associated to microglia activation (Guedes et al., 2013) and that miRNA(miR)-155 can contribute to neuroinflammation in AD (Guedes et al., 2014). Interestingly, upregulated miR-155 and miR-146a plus downregulated miR-124 were recently observed in microglia upon stimulation with lipopolysaccharide (LPS) (Cunha et al., 2016). Actually, these miRNAs are considered to modulate the inflammatory status (Olivieri et al., 2013b) and to be associated to microglia activation and polarization (Ponomarev et al., 2013).

Over the years several studies attempted the identification of microglia activation subtypes in several *in vitro* and *in vivo* models, as well as in AD brain autopsy specimens, trying to fit them into the described polarization schemes (Walker and Lue, 2015). Although the priming of microglia and the polarization into the M1 phenotype have been suggested by most of the works in AD (Heneka et al., 2015; Hoeijmakers et al., 2016), others also indicate increased expression of Arginase 1 (Colton et al., 2006) and co-expression of M1, M2a, M2b and M2c markers (Wilcock, 2012; Sudduth et al., 2013). Lately, five microglia morphological phenotypes (i.e., ramified, hypertrophic, dystrophic, rod-shaped and amoeboid) were identified in AD patient autopsied samples, together with an increased prevalence of dystrophic microglia in cases of dementia with Lewy bodies (Bachstetter et al., 2015). Contrasting results obtained so far derive from the diversity of the experimental models that are tentatively used to recapitulate the *in vivo* AD condition.

Relatively to microglia, *in vitro* cell models, either microglial cell lines, or primary microglia isolated from embryonic (Gingras et al., 2007) or neonatal animals (Floden and Combs, 2007), though largely used (Moussaud and Draheim, 2010), fail in mimicking adult behavior cells (Sierra et al., 2007). Furthermore, primary cultures of microglia were shown to change their activation profile according with the time in culture (Cristóvão et al., 2010). All of these features contribute to data inconsistency.

Since AD is considered an age-related disease, the use of aged animal models have been proposed (Bachstetter et al., 2015). However, a lot of problems must be considered. Actually, the need to wait for 2–3 years for animals aging to assess differences in cell function, and only in the survival population, together with a high result variability (Birch et al., 2014), have contributed to misunderstand many of the elderly processes and to failure in obtaining successful innovative strategic approaches to AD. Therefore, we hypothesized that our experimental model of *in vitro* aging microglia (Caldeira et al., 2014) would add additional information on the microglia phenotypes occurring in AD onset and later along the disease progression, while also allowing the work with aged microglia, once there are no processes to isolate degenerating microglia for experimentation (Njie et al., 2012).

In the present study we assumed that the recently isolated microglia maintained for 2 days *in vitro* (DIV) and the 16 DIV aged cultured microglia represent distinct cell populations that should react differently to the Aβ stressful stimulus. These subtypes may underlie diverse vulnerabilities along AD progression, from onset to late stages, and serve as models to better understand changes associated to cell malfunction by Aβ accumulation and by aging, not completely clarified so far. We considered that the 2 DIV young microglia phenotype of our previous study (Caldeira et al., 2014) mostly resemble the activation of the cell in the subacute inflammation state, while the 16 DIV aged cells recapitulate cells unable to mount an efficient response against a stressor stimulus. Hence, we aimed to examine the behavior of these two *in vitro* cultured microglia phenotypes, young/reactive (2 DIV) and aged/desensitized (16 DIV) cells, when facing a non-toxic mixture of Aβ_1–42_ oligomeric and fibrillar species at a concentration of 1000 nM. For that, we decided to assess cell morphology, phagocytic ability, migration capacity, autophagy and senescence markers, as well as a set of inflammatory-associated miRNAs, inflammatory cytokines, the alarmin high mobility group box 1 (HMGB1) protein, key regulatory receptors and inflammasome complex, together with matrix metalloproteinase 2 (MMP2) and MMP9 activation. Further, we evaluated gene expression of microglia phenotype M1 and M2 biomarkers and explored their subtype distribution.

Our results indicate that Aβ_1–42_, although prompting an acute inflammatory reaction, promote the switch of the activated microglia towards a miscellaneous polarized population, while eliciting microglia senescence and impairing phagocytosis in the 2 DIV *in vitro* microglia. Data also highlight the presence of an increased population of CD86+ microglia in the 16 DIV cells, whose expression is associated to cell plasticity and multipolar morphology. The number of CD86+ cells that increased in the presence of Aβ_1–42_, further suggests the simultaneous existence of pro- and anti-inflammatory phenotypes and a lower ability to mount immune and neuroprotective responses by the aged microglia. We believe that a better understanding on the significance of these two activated/dysfunctional cell states on AD pathogenesis will contribute to dissect microglial mechanisms in AD. If microglial diversity is confirmed in subsequent studies, different therapeutic approaches may be required to ensure effectiveness in a disease where personalized medicine and patient stratification are considered critical issues.

## Materials and Methods

### Animals

Animal care followed the recommendations of the European Convention for the Protection of Vertebrate Animals Used for Experimental and Other Scientific Purposes (Council Directive 86/609/EEC) and National Law 1005/92 (rules for protection of experimental animals). All animal procedures were approved by the Institutional animal care and use committee. Every effort was made to minimize the number of animals used and their suffering.

### Primary Culture of Microglia

Mixed glial cultures were prepared from 1 to 2 day-old CD1 mice, as previously described (McCarthy and de Vellis, 1980), with minor modifications (Gordo et al., 2006). Cells (4 × 10^5^ cells/cm^2^) were plated on uncoated 12-well tissue culture plates (with 18 mm coverslips) or 75-cm^2^ culture flasks in culture medium (DMEM-Ham’s F12 medium supplemented with 2 mM L-glutamine, 1 mM sodium pyruvate, 1% nonessential amino acids, 10% fetal bovine serum (FBS), and 1% antibiotic-antimycotic solution), and maintained at 37°C in a humidified atmosphere of 5% CO_2_.

Microglia were isolated as previously described (Saura et al., 2003). Briefly, after 21 days in culture, microglia were obtained by mild trypsinization with a trypsin-EDTA solution diluted 1:3 in DMEM-Ham’s F12 for 45–60 min. The trypsinization resulted in detachment of an upper layer of cells containing astrocytes, whereas microglia remained attached to the bottom of the well. The medium containing detached cells was removed and the initial mixed glial-conditioned medium was added. The use of this isolation procedure of mixed astrocyte-microglia cultures for 21 DIV cells allows a maximal yield and purity of microglia after trypsinization. In fact, astrocyte contamination was less than 2%, as assessed by immunocytochemical staining using a primary antibody against glial fibrillary acidic protein (GFAP) and a species-specific fluorescent-labeled secondary antibody. Neuronal contamination was 0%, as assessed by immunocytochemical staining with a primary antibody against microtubule-associated protein 2 (MAP2) followed by a species-specific fluorescent-labeled secondary antibody (Silva et al., 2010).

### Treatment of Microglia with a Mixture of Aβ_1–42_ Oligomers and Fibrils

Aβ_1–42_ peptide was diluted in DMEM-Ham’s F12 culture medium to a stock concentration of 111 μM and allowed to incubate for 24 h at 37°C to preaggregate the peptides, as formerly indicated (Hjorth et al., 2013). Cells were incubated with 50 nM and 1000 nM Aβ_1–42_, during 24 h, at 37°C, although the lower concentration was later abandoned in favor of the more consistent results obtained with the higher one. Cells incubated in the absence of Aβ_1–42_ were used as controls. We have previously observed that such Aβ_1–42_ solution was mainly constituted by large oligomers and fibrils with a small proportion of monomers and dimers, after a 24 h period of time (Falcão et al., 2017).

Isolated microglia were differentially aged in culture for 2 and 16 DIV in order to obtain two diverse microglia phenotypes, in accordance with our prior publication (Caldeira et al., 2014), and to assess whether they differentially respond to the Aβ stimulus. Actually, the 2 DIV cells represent an activated microglia subtype determined by the acute process of isolation, and the 16 DIV a more unresponsive/dormant subclass.

### Determination of Cell Death

To evaluate microglia cell death, we used phycoerythrin-conjugated annexin V (V-PE) and 7-amino-actinomycin D (7-AAD; Guava Nexin^®^ Reagent, #4500-0450, Merck Millipore, Billerica, MA, USA) to determine the percentage of viable, early-apoptotic and late-apoptotic/necrotic cells by flow cytometry. After incubation, plated microglia were trypsinized and added to cells in the incubation media, which were then stained with annexin V-PE and 7-AAD, following manufacturer’s instructions, and analyzed on a Guava easyCyte 5HT flow cytometer (Guava Nexin^®^ Software module, Millipore), as previously described (Barateiro et al., 2012). The three populations of cells that can be distinguished by this assay are the viable cells (annexin V-PE and 7-AAD negative), the early apoptotic cells (annexin V-PE positive and 7-AAD negative), and the cells in late stages of apoptosis or dead cells (annexin V-PE and 7-AAD positive).

### Cell Morphological Analysis

For morphological analysis, cells were fixed for 20 min with freshly prepared 4% (w/v) paraformaldehyde in phosphate buffer saline (PBS), and stained with a primary antibody against Ionized calcium binding adaptor molecule 1 (Iba1) (rabbit, 1:250; #019-19741, Wako Pure Chemical Industries Ltd, Osaka, Japan), and a secondary antibody Alexa Fluor 594 goat anti-rabbit (1:1000; #R37117, Invitrogen Corporation, Carlsbad, CA, USA). To identify the total number of cells, microglial nuclei were stained with Hoechst 33258 dye (Sigma Chemical Co., St. Louis, MO, USA). Fluorescence was visualized using an AxioCam HRm camera adapted to an AxioSkope^®^ microscope (Zeiss, Germany). Pairs of U.V. and red-fluorescence images of 10 random microscopic fields (original magnification: 400×) were acquired per sample. To characterize microglia morphology we used the particle measurement feature in ImageJ (1.47v, USA) to automatically obtain the 2D area, perimeter, circularity, and Feret’s diameter of single microglia. Circularity of microglia was obtained by the formula: Circularity = 4π (area/perimeter^2^). A circularity value of 1.0 indicates a perfectly circular cell, and values near zero indicate elongated and ramified microglia. Feret’s (maximum) diameter, a measure of cell length, is the highest distance between any two points along the cell perimeter.

### Evaluation of MMP2 and MMP9 Activities

Assessment of MMP2 and MMP9 activities in the extracellular medium was based on their ability to degrade gelatin. For that, 20 μl of incubation medium was resolved using a SDS-PAGE zymography of 0.1% gelatin—10% acrylamide gel. After electrophoresis, gels were washed for 1 h with 2.5% TritonX-100 (in 50 nM CaCl_2_; 1 μM ZnCl_2_) to remove SDS and renature MMP species in the gel. To allow gelatin degradation by MMPs, gels were incubated overnight, at 37°C, in the developing buffer (50 mM Tris pH 7.4; 5 mM CaCl_2_; 1 μM ZnCl_2_). For enzyme activity analysis, the gels were stained with 0.5% Coomassie Brilliant Blue R-250 and distained in 30% ethanol/10% acetic acid/H_2_O. Gelatinase activity, detected as a white band on a blue background, was measured by computerized image analysis (Image Lab™ Software 3.0, Bio-Rad Laboratories Inc., Grand Junction, CO, USA) and normalized to cellular protein content (Silva et al., 2010). Activities of MMP2 and MMP9 were distinguished thanks to their different relative molecular weight, i.e., MMP2 of 72 kDa and MMP9 of 92 kDa (Frankowski et al., 2012).

### Assessment of Microglia Autophagy

Autophagy was determined by immunocytochemistry based on the punctate pattern of the microtubule-associated-protein-light-chain-3 (LC3) and Western Blot detection of Beclin-1 bands, as previously described (Caldeira et al., 2014). For immunocytochemistry, cells were fixed as above, and we used rabbit anti-LC3 protein (1:500; #2775S, Cell Signaling Technology Inc., Danvers, MA, USA) as a primary antibody, and Alexa Fluor 488 goat anti-rabbit (1:1000; #A-11034, Invitrogen Corporation) as the secondary one. Nuclei were stained with Hoechst 33258 dye. Fluorescence was visualized and images acquired as above mentioned. Increased LC3 autophagosome puncta indicates induced autophagy. For Western Blot, cell extracts were separated in sodium dodecyl sulfate-polyacrylamide gel electrophoresis and transferred to a nitrocellulose membrane. Immunoblots were performed as usual in our lab using as primary antibodies mouse anti-Beclin-1 (1:500; #MABC34, MerckMillipore) and mouse anti-β-actin (1:5000; #A5441, Sigma), followed by respective secondary horseradish peroxidase-labeled antibodies. Results were normalized to β-actin expression and expressed as fold vs. 2 DIV non-treated (control) cells.

### Determination of Microglia Senescence

Microglial senescence was determined using the Cellular senescence assay kit (Millipore) that evaluates the activity of senescence-associated β-galactosidase (SA-β-gal), according to manufacturer instructions. Microglial nuclei were counterstained with hematoxylin. Light microscopy images of 10 random microscopic fields (original magnification: 400×) were acquired per sample using a Leica DC 100 camera (Leica, Wetzlar, Germany) adapted to an Axioskop microscope (Zeiss). Turquoise blue stained microglia were considered as senescent cells and their percentage calculated relatively to the total number of cells. Protein expression of ferritin was performed by Western Blot as above, using the primary antibody rabbit anti-FHT1 (1:500, #4393, Cell Signaling Technology Inc., Danvers, MA, USA).

### Microglia Migration Assessment

Cell migration is often assessed with the classic Boyden Chamber assay, where cells loaded in the upper well are allowed to migrate through filter pores to the lower compartment of the chamber. Assays were performed in a 48-well microchemotaxis Boyden chamber (Neuro Probe, Gaithersburg, MD, USA), as previously described (Miller and Stella, 2009), with minor modifications. The bottom wells were filled with control medium (DMEM-Ham’s F12) and Aβ (1000 nM) to evaluate the ability of microglia to move towards Aβ. ATP (10 μM) applied in the lower well was also used as a positive control for microglia migration, since it is a known chemoattractant for microglia. The 8 μm diameter polycarbonate membranes with polyvinylpyrrolidone (PVP) surface treatment was equilibrated in control medium and after chamber set up, 50 μl of cell suspension containing 2 × 10^4^ was added to each top well. After 6 h incubation in a CO_2_ incubator at 37°C to allow microglia migration, membrane was fixed with ice-cold methanol and cells stained with 10% Giemsa (Sigma) in PBS. Non-migrated cells on the upper side of the membrane were wiped off with a filter wiper. The rate of migration was determined by counting the cells on the lower membrane surface, using 10 microscopic fields (original magnification: 100×). Images were acquired with a Leica DFC490 camera adapted to an AxioSkope HBO50 microscope. For each experiment, data from at least three wells per condition were acquired.

### Evaluation of Microglia Phagocytic Ability

The efficiency of the microglial phagocytosis was assessed by counting the number of ingested beads per cell, considering the total number of cells, to obtain the average amount of ingested beads per cell, as well as by the percentage of cells phagocytosing less than 5, 5–10, or more than 10 beads. The method consists in incubating the primary microglial cultures, differentially aged in culture with 0.0025% (w/w) of 1 μm fluorescent latex beads (Sigma) for 75 min at 37°C. Thereafter, cells were fixed with freshly prepared 4% (w/v) paraformaldehyde in PBS. Microglia were stained for Iba1 and nuclei counterstained with Hoechst 33258 dye. Fluorescence was visualized and acquired as above mentioned.

### Determination of Aβ in Cells and Lysates

Aβ in cell lysates was determined by Western Blot using anti-Aβ clone W0-2 (1:500, #MABN10, MerkMillipore) as the primary antibody. Extracellular deposition of Aβ was observed by immunocytochemistry using antibodies against Iba1 to detect microglia cell body and Aβ clone W0-2 for amyloid deposits. Nuclei were counterstained with Hoechst 33258 dye.

### Detection of Specific miRNA Expression Changes

To evaluate changes in miRNAs with a crucial role in microglia function/dysfunction and polarization, we assessed the expression of miR-124, miR-155 and miR-146a by quantitative realtime PCR (qRT-PCR). Total RNA was extracted from primary microglia cultures using the miRCURY™ Isolation Kit-Cells (Exiqon, Denmark), according to the manufacturer’s recommendations for cultured cells. Briefly, after cell lysis, the total RNA was adsorbed to a silica matrix, washed with the recommended buffers and eluted with 35 μl RNase-free water by centrifugation. After RNA quantification, conversion to cDNA was performed using the universal cDNA Synthesis Kit (Exiqon) and 20 ng total RNA according to the following protocol: 60 min at 42°C followed by heat-inactivation of the reverse transcriptase for 5 min at 95°C. qRT-PCR was performed in an 7300 Real time PCR System (Applied Biosystems, Madrid, Spain) using 96-well plates. For miRNA quantification the miRCURY LNA™ Universal RT microRNA PCR system (Exiqon) was used in combination with pre-designed primers (Exiqon), which are represented in Supplementary Table S1 (Supplementary Material), using SNORD110 as reference gene. The reaction conditions consisted of polymerase activation/denaturation and well-factor determination at 95°C for 10 min, followed by 50 amplification cycles at 95°C for 10 s and 60°C for 1 min (ramp-rate 1.6°/s). The miRNA fold increase/decrease with respect to control samples was determined by the Pfaffl method, taking into consideration different amplification efficiencies of miRNAs in all experiments. The amplification efficiency for each target was determined according to the formula: *E* = 10^(−1/S)^ − 1, where S is the slope of the obtained standard curve.

### Gene Expression Profiling

qRT-PCR was performed for mRNA expression, as usual in our laboratory (Barateiro et al., 2013). Total RNA was extracted from microglia using TRIzol^®^ (Life Technologies, Inc., Grand Islands, USA), according to manufacturer’s instructions. Total RNA was quantified using Nanodrop ND-100 Spectrophotometer (NanoDrop Technologies, Wilmington, DE, USA). Aliquots of 0.5 μg of total RNA were treated with DNase I and then reverse transcribed to produce cDNA using oligo-dT primers and SuperScript II Reverse Transcriptase under the recommended conditions. qRT-PCR was performed on a 7300 Real time PCR System (Applied Biosystems) using a SYBR Green qPCR Master Mix (Fermentas, Ontario, Canada), and β-actin as an endogenous control to normalize the expression level of the different genes. Primer sequences that were used are indicated in Supplementary Table S2 (Supplementary Material). PCR was performed in 96 well plates and triplicate analysis was accomplished for each sample. No-template control was included for each amplificate. Cycling conditions were 94°C for 3 min followed by 40 cycles at 94°C for 0.15 min, 62°C for 0.2 min and 72°C for 0.15 min. A melt-curve analysis was used to verify the specificity of the amplification, immediately after the amplification protocol. Non-specific products of PCR were not found in any case. Relative mRNA concentrations were calculated using the Pfaffl modification of the ∆∆CT equation (CT, cycle number at which fluorescence passes the threshold level of detection), considering the efficiencies of individual genes. The results were normalized to β-actin in the same sample, and the initial amount of the template of each sample determined as relative expression by the formula 2-∆∆CT. ∆CT in each sample derives from the difference between the mean CT value of each gene and the mean CT value of β-actin. ∆∆CT of one sample is the difference between its ∆CT value and ∆CT of the selected reference, in our case the 2 DIV non-treated (control) cells.

### Identification of CD11b and CD86 Microglia Populations by Flow Cytometry

Cells were resuspended in PBS and kept in the flow buffer (PBS plus 2% FBS and 0.02% sodium azide). To prevent non-specific binding, cells were incubated for 20 min with CD16/CD32 (1:100) to block Fc receptors, at 4°C. Afterwards, cell suspension was incubated with the fluorescent labeled antibodies (CD11b PerCp-Cy5, CD45 PE and CD86 Bio-SAV PE) for 30 min, at 4°C (1:100). Following the incubation, cells were washed with the flow buffer, incubated with streptavidin (1:100) for the CD86 Bio-SAV PE antibody during 30 min, and then resuspended in the flow buffer. Expression of surface antigens was evaluated using the BD FACSCalibur flow cytometer (Becton Dickinson, San Jose, CA, USA) and data analyzed using the FlowJo software.

### Statistical Analysis

Results of at least four different experiments are expressed as mean ± SEM. Significant differences between control and Aβ treated groups were determined by *t*-test. To compare the effects of Aβ treatment and microglia differentially aged in culture, two-way analysis of variance (ANOVA) was performed using GraphPad Prism^®^ 5.0 (GraphPad Software, San Diego, CA, USA). Statistical significance was considered for *p* < 0.05.

## Results

### Microglia Treated with Aβ do Not Show Age-Dependent Changes in Cell Viability

In the present work we used our established model of reactive and aged-like microglia phenotypes, in which the microglial cells are separated from mixed cultures with astrocytes and maintained for 2 DIV and 16 DIV in culture, respectively (Caldeira et al., 2014). Our main interest was to see whether activated/young cells would respond differently from the aged cells to Aβ. For that, we decided to incubate the cells with a mixture of Aβ oligomers and fibrils, as previously used in the N9 microglial cell line (Falcão et al., 2017), to recapitulate the neuropathology of AD associated to the different activation processes of microglia by such species (Sondag et al., 2009). Although we have tested 50 nM and 1000 nM Aβ concentrations, as in our earlier study (Falcão et al., 2017), the effects obtained were more reliable for the highest level, reason why we decided to only proceed with Aβ 1000 nM for a 24 h treatment period.

As a first step, and in order to guarantee that the viability of the aged-cultured microglia (16 DIV) was equivalent to that of the acutely (2 DIV) isolated cells, we assessed the percentage of viable, early-apoptotic and late-apoptotic/necrotic cells by flow cytometry in both adherent and detached cells, as described in “Materials and Methods” Section. As depicted in Table 1, although a slight increase was observed in the number of cells showing late-apoptosis/necrosis upon treatment with Aβ, namely in the 16 DIV microglia, the lack of significance of such effects point out no direct influence of cell viability differences on the events presented in the following sections.

**Table 1:** Microglia viability is not altered by amyloid-β (Aβ) treatment.

	2 DIV		16 DIV	
	Control	Aβ	Control	Aβ
Viable	73.6 (± 9.1)	65.6 (± 6.7)	70.7 (± 7.1)	66.5 (± 5.7)
Early-apoptosis	18.8 (± 8.6)	17.8 (± 4.8)	21.7 (± 8.1)	21.6 (± 5.3)
Late-apoptosis/necrosis	2.9 (± 1.4)	3.7 (± 1.4)	5.8 (± 1.0)	9.1 (± 2.4)

*Results are expressed as percentage of total number of cells. Values are mean ± SEM from at least four independent experiments. Microglial cells were kept in culture for 2 days in vitro (DIV) and 16 DIV and treated with Aβ at 1000 nM for 24 h. The percentage of viable microglia and microglia in early- and late-apoptosis/necrosis was determined by flow cytometer with phycoerythrin-conjugated annexin V (annexin V-PE) and 7-amino-actinomycin D (7-AAD). The three populations were distinguished as follows: viable cells (annexin V-PE and 7-AAD negative), early apoptotic cells (annexin V-PE positive and 7-AAD negative), and late stages of apoptosis or dead cells (annexin V-PE and 7-AAD positive)*.

### Young and Aged Cultured Microglia Show Soma Enlargement by Aβ treatment

Characterization of microglia morphometric features has been associated to cell polarization and activation state (Torres-Platas et al., 2014). While the ramified morphology relates with the surveilling cell, the amoeboid microglia are associated with activation and believed to favor phagocytosis and mobility (Lull and Block, 2010).

**Figure 1:**
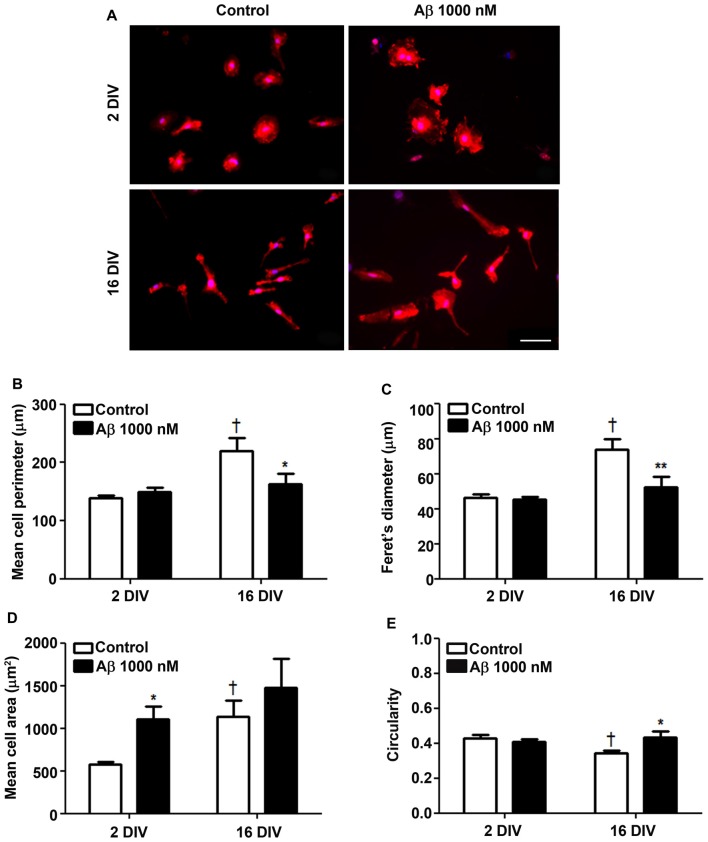
Reactive and aged cultured microglia display soma enlargement and amoeboid morphology after treatment with amyloid-β (Aβ) peptide. Microglia that were kept in culture for 2 and 16 days *in vitro* (DIV) were treated with 1000 nM Aβ for 24 h. Cells were immunostained for Iba1 and characterized for their morphometric features. **(A)** Representative images show increased ramification by age, which was counteracted by Aβ exposure. Microglia perimeter **(B)**, Feret’s diameter **(C)**, area **(D)** and circularity values **(E)** were measured using ImageJ software and expressed in graph bars as mean ± SEM. Cultures, *n* = 4 per group. Two-way analysis of variance (ANOVA; *Post hoc* Bonferroni test): *p < 0.05 and **p < 0.01 vs. respective non-treated Control; ^†^*p* < 0.05 vs. 2 DIV cells; **(B)** DIV × Aβ interaction *F*_(4.74)_, *p* < 0.05; **(C)** DIV × Aβ interaction *F*_(5.27)_, *p* < 0.05; **(E)** DIV × Aβ interaction *F*_(5.14)_, *p* < 0.05. Scale bar equals 50 μm.

The cells cultured for 2 DIV (young/reactive) showed a predominant amoeboid morphology, resulting from the acutely isolation protocol that causes the activation of microglia (Figure 1A), as previously demonstrated (Caldeira et al., 2014). When aged in culture for 16 DIV, the cells exhibited polarized and ramified populations, including rod-like and bipolar morphologies, determining increased cell perimeter and Feret’s maximum diameter (1.6-fold, *p* < 0.05; Figures 1B,C). Curiously, while Aβ treatment of 2 DIV microglia promoted a prevalent ovoid shape with an enlarged cell area (1.9-fold, *p* < 0.05; Figure 1D), that of 16 DIV led to heterogeneous morphologies, with polarized microglia bearing one and two large processes, or determined a large lamellipodia with a thin process. In this case the cells showed a reduction in cell perimeter (0.7-fold, *p* < 0.05) and in Feret’s maximum diameter (interaction between DIV and Aβ treatment of *F*_(4.74)_ and *F*_(5.27)_, respectively, *p* < 0.05), as well as an increased circularity (1.2-fold, *p* < 0.05; interaction between DIV and Aβ treatment of *F*_(5.14)_, *p* < 0.05; Figure 1E). These morphometric alterations suggest that both young and aged cells suffer an increase in soma volume, although the process shortening after Aβ treatment was less notorious in aged cells. To what extent these changes represent an equally activated microglia or a different functional state will be examined in the following sections.

### Aβ Diversely Activates MMP2 and MMP9 in Reactive and Aged Cultured Microglia

**Figure 2:**
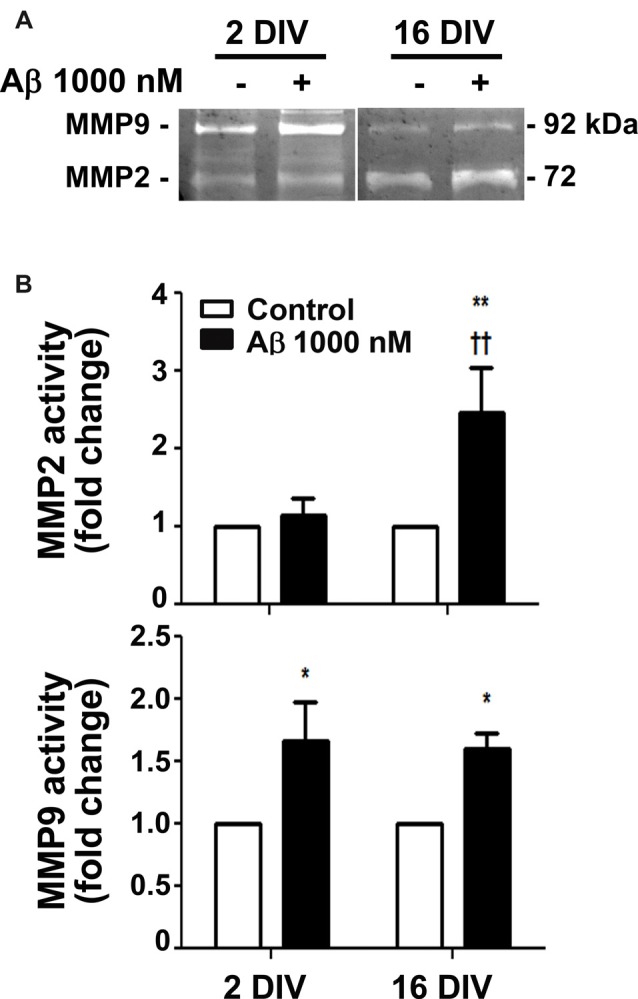
Release of matrix metalloproteinase 2 (MMP2) and MMP9 is differently induced by amyloid-β (Aβ) peptide in reactive and aged cultured microglia. Microglia that were kept in culture for 2 and 16 days *in vitro* (DIV) were treated with 1000 nM Aβ for 24 h. Activities of MMP2 and MMP9 were evaluated by gelatin zymography.** (A)** Representative images of zymography gels. **(B)** Results are expressed in graph bars as mean ± SEM. Cultures, *n* = 4 per group. Two-way ANOVA (*Post hoc* Bonferroni test): *p < 0.05 and **p < 0.01 vs. respective non-treated Control; ^††^*p* < 0.01 vs. 2 DIV; **(B)** DIV × Aβ interaction *F*_(4.39)_, *p* < 0.05.

MMPs were shown to be important for Aβ degradation (Qiu et al., 1997), and MMP3, MMP12 and MMP13 to be activated by Aβ (Ito et al., 2007). Intriguingly, MMP2 and MMP9 revealed to be differently activated in diverse experimental and animal models, as well as in AD patients, and to be related with the aggravation of AD disease (Brkic et al., 2015). We observed that Aβ triggered an increased activation of both MMP2 and MMP9 in the aged cells (2.4- and 1.5-fold, *p* < 0.01 and *p* < 0.05, respectively; interaction between DIV and Aβ treatment for MMP2 *F*_(4.39)_, *p* < 0.05), while only stimulated MMP9 in the young reactive microglia (1.7-fold, *p* < 0.05; Figure 2). Data suggest that aged microglia may use these MMPs to degrade Aβ and inhibit its accumulation. However, the dual roles of MMPs complicate the understanding of the significance of such results relatively to their potential beneficial or harmful effects in AD (Wang et al., 2014).

### Aβ Induces Age-Dependent Changes in Autophagy-Related Beclin-l Gene and LC3 Puncta

**Figure 3:**
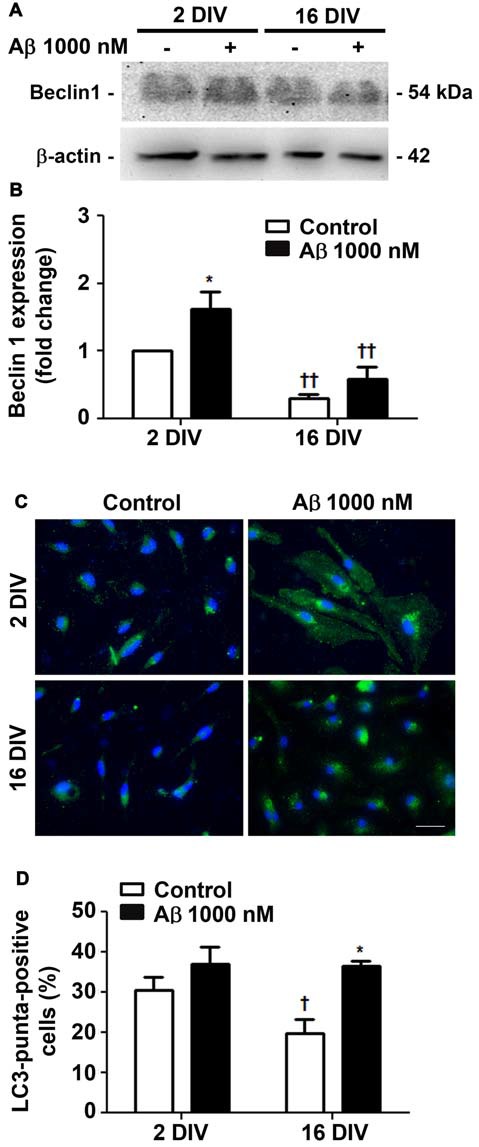
Autophagy is differently promoted by amyloid-β (Aβ) peptide in reactive and aged cultured microglia. Microglia that were kept in culture for 2 and 16 days *in vitro* (DIV) were treated with 1000 nM Aβ for 24 h. Total cell lysates were analyzed for the presence of Beclin 1. **(A)** Representative images of Beclin 1 protein expression. **(B)** Results of densitometric analysis of Beclin 1 blots are expressed in graph bars as mean ± SEM. Microtubule-associated- protein-light-chain-3 (LC3)-positive puncta cells were detected by immunostaining for LC3. **(C)** Representative images of immunocytochemistry for LC3 (green) and nuclei staining (blue). Scale bar equals 50 μm. **(D)** Percentage of cells showing LC3-positive puncta are expressed in graph bars as mean ± SEM. Cultures, *n* = 4 per group. Two-way ANOVA (*Post hoc* Bonferroni test): *p < 0.05 vs. respective non-treated Control; ^†^*p* < 0.05 and ^††^*p* < 0.01 vs. 2 DIV.

Autophagy, or cellular self-digestion, is a highly regulated and evolutionarily conserved process that was shown to be impaired in AD (Zare-Shahabadi et al., 2015). We first evaluated microglia autophagic capacity by assessing the expression of Beclin-1, a protein known to be recruited to phagosomal membranes, and to participate in the early stages of autophagy and LC3-associated phagocytosis (Chifenti et al., 2013). We observed that Beclin-1 was upregulated by Aβ in young/activated 2 DIV cells (1.6-fold, *p* < 0.05, Figures 3A,B), while was downregulated in aged 16 DIV cells and only suffered a slight and not significant increase upon Aβ stimulation. Based on the importance of LC3 processing for autophagosome formation, we next determined LC3-positive puncta indicative of such formation/accumulation (Klionsky et al., 2016). As shown Figures 3C,D, Aβ only slightly increased the number of 2 DIV cells presenting LC3-positive puncta. Conversely, reduced autophagy in aged cells was upregulated under Aβ treatment to values closely resembling those of 2 DIV cells. Together, these results suggest that Aβ promotes the formation of autophagosomes, which turnover may be impaired in aged microglia and contribute to Aβ accumulation within the cells. In addition, Aβ switches the reactive 2 DIV microglia towards a senescent-like cell phenotype, perhaps compromising the response to stressors.

### Aβ Upregulates Senescence-Associated Biomarkers in 2 DIV Microglia Towards Values of 16 DIV Cells

**Figure 4:**
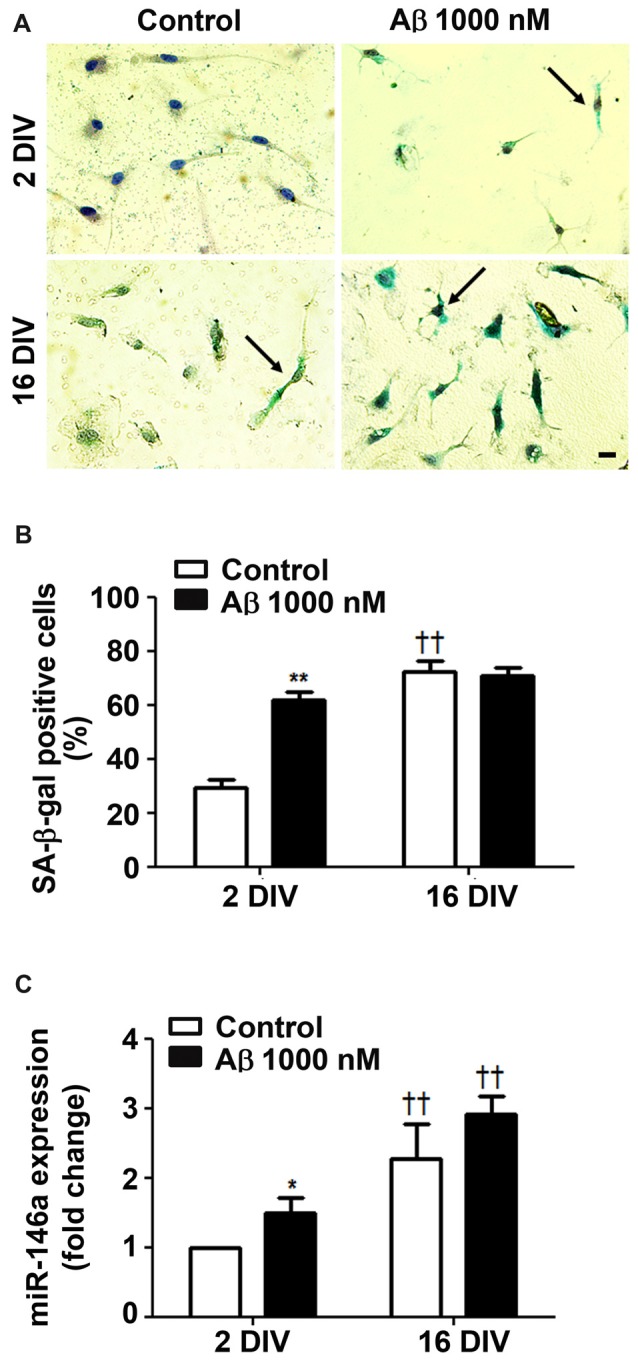
Amyloid-β (Aβ) peptide promotes cell senescence in reactive cultured microglia. Microglia that were kept in culture for 2 and 16 days *in vitro* (DIV) were treated with 1000 nM Aβ for 24 h. Activity of senescence-associated β-galactosidase (SA-β-gal) was determined using a commercial kit, and SA-β-gal-positive cells were counted.** (A)** Representative images of cells showing blue turquoise SA-β-gal staining. Scale bar equals 20 μm. **(B)** Percentage of cells showing SA-β-gal-positive staining are expressed in graph bars as mean ± SEM. **(C)** MicroRNA (miR)-146a expression was evaluated by Real-Time PCR. Results are expressed in graph bars as mean ± SEM. Cultures, *n* = 4 per group. Two-way ANOVA (*Post hoc* Bonferroni test): *p < 0.05 and **p < 0.01 vs. respective non-treated Control; ^††^*p* < 0.01 vs. 2 DIV cells; **(B)** DIV × Aβ interaction *F*_(28.1)_, *p* < 0.01.

Since Beclin-1 levels were shown to decline by aging (Shibata et al., 2006), we next assessed the cells that positively stained for SA-β-gal, a biomarker of cellular senescence (Sikora et al., 2011). As shown in Figures 4A,B, treatment of young/reactive microglia with Aβ increased the number of SA-β-gal positive cells (2.1-fold, *p* < 0.05) to values near to those of 16 DIV aged cells (interaction between DIV and Aβ treatment *F*_(28.1)_, *p* < 0.01), independently of being treated or not with Aβ.

Ferritin was found most related with proliferative microglia in AD hippocampus from patients (Grundke-Iqbal et al., 1990). However, a subpopulation of dystrophic microglia were also shown to be positive for ferritin (Lopes et al., 2008). Indeed, we have observed that both 2 DIV and 16 DIV Aβ-treated microglia showed increased levels of ferritin, although the expression was less predominant in the aged cells (data not shown). Other studies also observed a decrease in ferritin accumulation with age in substantia nigra (Walker et al., 2016), which was referred to compromise cell resistance to reactive oxygen species (ROS) (Yang et al., 2013). To further assess if Aβ induces a senescent-like response in 2 DIV microglial cells, we decided to evaluate miR-146a expression in the differently *in vitro* aged microglial cells. Actually, besides its numerous described functions and targets (Cardoso et al., 2016), miR-146a was reported to contribute to age-related dysfunction of macrophages (Jiang et al., 2012), and to loss of mitochondrial integrity and function in aged cells (Rippo et al., 2014). As anticipated, miR-146a increased expression was observed in the 2 DIV microglia treated with Aβ, although not reaching the values of 16 DIV cells (Figure 4C). Overall, Aβ switches the reactive 2 DIV microglia towards a senescent-like cell phenotype with potential negative consequences to a stress response.

### Aβ Impairs Microglia Migration Ability in the Aged Cultured Cells

**Figure 5:**
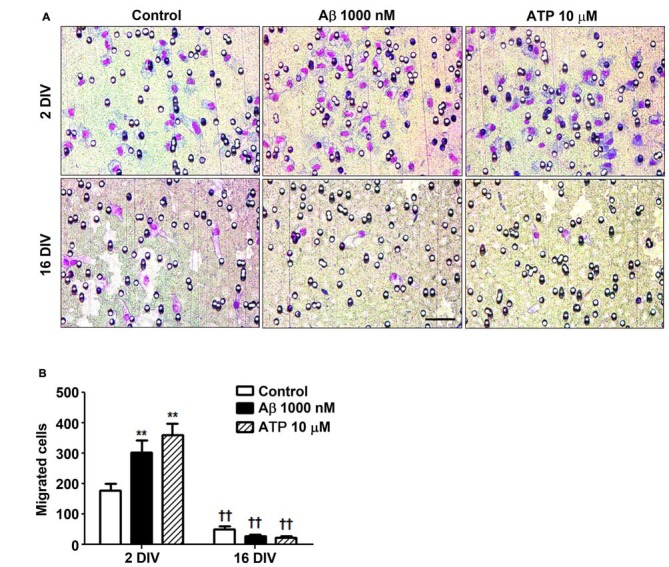
Reactive cultured microglia show increased ability to migrate towards amyloid-β (Aβ) peptide and ATP, while aged microglia are immotile and unresponsive to such chemoattractants. Microglia were kept in culture for 2 and 16 days *in vitro* (DIV) and cellular chemotactic migration to 1000 nM Aβ and 10 μM ATP (positive chemotactic control) was evaluated after 6 h incubation using the Boyden chamber method. **(A)** Representative images of cells that have migrated to Aβ and ATP. Scale bar equals 50 μm. **(B)** Number of migrated cells was counted and results are expressed in graph bars as mean ± SEM. Cultures, *n* = 4 per group. Two-way ANOVA (*Post hoc* Bonferroni test): **p < 0.01 vs. respective non-treated Control; ^††^*p* < 0.01 vs. 2 DIV cells; **(B)** DIV × Aβ interaction *F*_(4.36)_, *p* < 0.05.

Microglia important functions in the CNS include migration dynamics, synaptic pruning and phagocytosis of neuronal cells and their debris (Xavier et al., 2014; Zhang et al., 2016). Cell migration can be triggered by several chemoattractants, including ATP that when released by damaged neurons acts on P2Y_12_ and P2X_4_ receptors in microglia stimulating their migration (Miller and Stella, 2009). Our data showed that young microglia exhibited higher migration ability than the older cells (Figure 5) and that, in contrast with those, positively respond to Aβ (1.7-fold, *p* < 0.01) and ATP chemotactic signals (2.0-fold, *p* < 0.01). These findings besides indicating that aged cells are in a dormancy-like state relatively to their capacity of migration, when compared with the 2 DIV cells, also demonstrate their unresponsiveness to chemoattractants, including Aβ, that may further compromise the phagocytic activity of 16 DIV microglia.

### Aβ Impairs Microglia Phagocytic Ability Mainly in the Reactive Cultured Microglia

**Figure 6:**
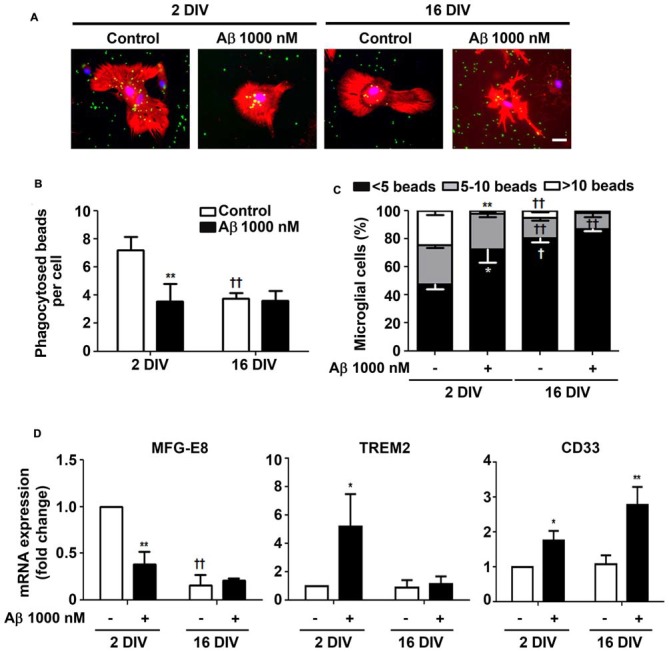
Ability of microglia to phagocytose is reduced by amyloid-β (Aβ) peptide mainly in the reactive cultured cells. Microglia that were kept in culture for 2 and 16 days *in vitro* (DIV) were treated with 1000 nM Aβ for 24 h. Phagocytic capacity was assessed after 75 min incubation with fluorescent latex beads. **(A)** Representative images of microglia immunostained for Iba1 (red) and stained with Hoechst for nuclei staining (blue) containing phagocytosed fluorescent latex beads (green). Scale bar equals 50 μm. **(B)** Number of phagocytosed beads per cell and **(C)** number of microglial cells phagocytosing less than 5, 5–10 and more than 10 beads was counted. **(D)** Expression of milk fat globule-EGF factor 8 protein (MFG-E8), triggering receptor expressed on myeloid cells 2 (TREM2) and CD33, associated to microglial phagocytosis, was evaluated by Real-Time PCR. Results are expressed in graph bars as mean ± SEM. Cultures, *n* = 4 per group. Two-way ANOVA (*Post hoc* Bonferroni test): *p < 0.05 and **p < 0.01 vs. respective non-treated Control; ^†^*p* < 0.05 and ^††^*p* < 0.01 vs. 2 DIV; **(D)** MFG-E8: DIV × Aβ interaction *F*_(13.9)_, *p* < 0.01.

Microglia phagocytosis is an important protective role for the efficient elimination of apoptotic cells and for neuronal circuitry reshape (Xavier et al., 2014). Our results corroborate previous data demonstrating a reduced phagocytosis by *in vitro* aged microglia (Caldeira et al., 2014). Here, we showed that 24 h incubation of reactive microglia with Aβ led to 2-fold reduction (*p* < 0.01) in the number of phagocytosed beads per cell, approaching the values obtained for 16 DIV cells, treated or not with Aβ, as depicted in Figures 6A,B. In addition, the number of 2 DIV cells able to engulf more than 10 beads was dramatically decreased upon Aβ interaction (10-fold reduction, *p* < 0.01, Figure 6C). Interestingly, the diminished number of 16 DIV cells phagocytosing 5–10 beads decreased even more upon Aβ treatment. These results suggest that Aβ reduces 2 DIV microglia phagocytosis towards the levels of aged/unresponsive microglia. We may conclude that despite the ability to migrate to sites of Aβ deposition the young/activated microglia lose their phagocytic capacity when facing Aβ, at least in primary cultures.

Phagocytosis may be mediated by several pathways. We next decided to explore milk fat globule factor-EGF factor 8 protein (MFG-E8) expression, a key factor involved in the phagocytosis of apoptotic cells, such as neurons (Fuller and Van Eldik, 2008; Liu et al., 2013). Following the same protocol as above, we tested whether Aβ interfered with the expression of MFG-E8 by 2 DIV and 16 DIV microglia. Interestingly, we observed that the levels of MFG-E8 suffered more than a 2-fold reduction (*p* < 0.01) in the 2 DIV microglia (Figure 6D). As before, the aged cells showed downregulated expression of this phagocytic-related protein, which was sustained in the presence of Aβ (significant interaction between DIV and Aβ treatment *F*_(13.92)_, *p* < 0.01), thus reinforcing their unresponsive nature. To next assess the expression of cell membrane surface receptors associated to phagocytosis, we evaluated the triggering receptor expressed on myeloid cells 2 (TREM2) and the type 1 transmembrane protein CD33. Both are members of the sialic acid-binding immunoglobulin-like lectins (Siglecs) and are expressed by immune cells. TREM2 is involved in the phagocytosis of damaged cells and showed to reduce the inflammatory response (Walter, 2016). Most attractively, heterozygous rare variants in TREM2 have been associated with a significant increase in the risk of AD emergence (Guerreiro et al., 2013), while TREM2 deficiency in an AD animal model, the 5xFAD, increased cerebral Aβ accumulation (Wang Y. et al., 2015). Expression of TREM2 mRNA was markedly increased in 2 DIV microglia upon Aβ exposure (5.2-fold, *p* < 0.05), as depicted in Figure 6D. However, unchanged values upon Aβ treatment were observed in 16 DIV microglia.

CD33 gene is considered a risk factor for AD and increased number of CD33-immunoreactive microglia were shown to correlate with insoluble Aβ42 levels and plaque burden in AD brain (Griciuc et al., 2013). Actually, increased expression of CD33 determines an impairment in microglia-mediated clearance of Aβ (Jiang et al., 2014). Our data revealed an increased expression of CD33 in 2 DIV microglia (1.7-fold, *p*<0.05), which was intensified in 16 DIV cells (2.8-fold, *p*<0.01; Figure 6D), thus reinforcing previous results showing a decreased microglial phagocytic ability towards Aβ species.

To further explore differences in Aβ phagocytosis between young/reactive and aged/unresponsive microglia, we evaluated Aβ species in cell lysates. As depicted in Supplementary Figure S1A, the 2 DIV microglia revealed a higher content of Aβ, namely of dimers and monomers, as compared to 16 DIV microglia that seem to contain increased oligomers. Aβ-immunostaining (Supplementary Figure S1B) confirmed the increased uptake of this peptide by young microglia. Although also detected in 16 DIV/aged microglia, the majority of deposits revealed to be localized outside the cells. Overall, our results indicate that young/reactive microglia attempt to phagocytose Aβ, based on the elevation of TREM2 expression, but that increased CD33 expression may counteract this feature. Deposits of Aβ surrounding the 16 DIV cells and marked expression of CD33 in 16 DIV microglia confirm the low ability of these cells to phagocytose Aβ.

### Aβ Reduces the Expression of Inflammatory-Related miR-155 and miR-124 in 2 DIV Microglia

**Figure 7:**
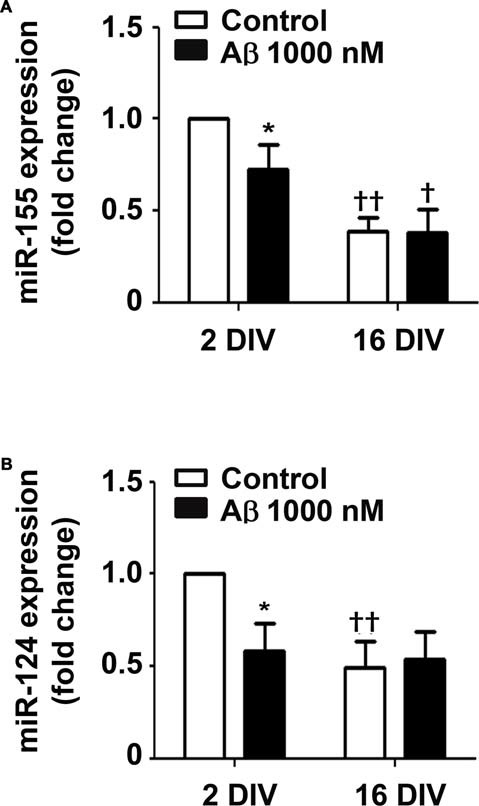
Amyloid-β (Aβ) peptide decreases the expression of miRNA (miR)-155 and miR-124 in the reactive cultured microglia toward that of 16 days *in vitro* (DIV) cells. Microglia that were kept in culture for 2 and 16 DIV were treated with 1000 nM Aβ for 24 h. **(A)** Expression of M1/pro-inflammatory-related miR-155 and of **(B)** M2/anti-inflammatory-related miR-124 was evaluated by Real-Time PCR. Results are expressed in graph bars as mean ± SEM. Cultures, *n* = 4 per group. Two-way ANOVA (*Post hoc* Bonferroni test); *p < 0.05 vs. respective non-treated Control; ^†^*p* < 0.05 and ^††^*p* < 0.01 vs. 2 DIV cells.

Recent studies indicate that miR-155 and miR-124a regulate T-cell functions during inflammation (Heyn et al., 2016). Both miRNAs are directly involved in microglia polarization, where miR-124 is considered to be associated with an anti-inflammatory M2 phenotype, and miR-155 as having a determinant role in microglia activation toward the M1 phenotype (Ponomarev et al., 2013). To gain insight into the Aβ-induced alterations in microglia polarization we assessed the expression of these inflammation-related miRNAs in the 2 DIV and 16 DIV microglia (Figure 7). Interestingly, while we obtained a downregulated expression of both miR-155 and miR-124 by Aβ treatment in the 2 DIV microglial cells (0.7-fold and 0.6-fold, respectively, *p* < 0.05), no changes were observed in the aged cultured microglia, which basal levels were already inferior to the 2 DIV control cells (~0.5-fold, *p* < 0.01) attesting a dormancy-like behavior of such cells. These results suggest that Aβ counteracts either M2 (low miR-124) or M1 (low miR-155) polarization in the 2 DIV microglia already activated by the isolation procedure. Thus, presence of mixed subpopulations and less responsive microglia subtypes following Aβ interaction should be envisaged as a consequence of this noxious stimulus.

### 16 DIV Cells Only React to Aβ Stimulus by Increasing the Expression of TNF-α and IL-1β, while the 2 DIV Microglia Show a Larger and More Intense Spectrum of Activation

**Figure 8:**
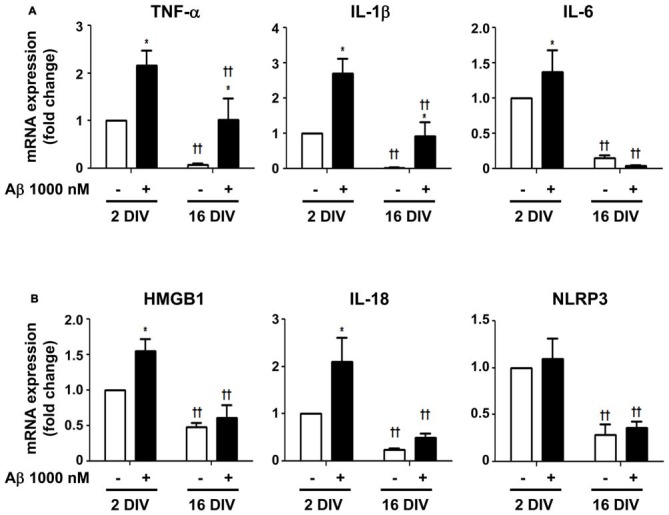
Increased expression of inflammatory mediators in microglia treated with amyloid-β (Aβ) peptide is more evident in the reactive cultured cells. Microglia that were kept in culture for 2 and 16 days *in vitro* (DIV) were treated with 1000 nM Aβ for 24 h. **(A)** Expression of inflammatory cytokines [e.g., tumor necrosis factor-α (TNF-α), interleukin (IL)-1β and IL-6] and of **(B)** inflammasome-related proteins [e.g., high-mobility group protein B1 (HMGB1), IL-18 and NOD-like receptor family pyrin domain containing 3 (NLRP3)] were evaluated by Real-Time PCR. Results are expressed in graph bars as mean ± SEM. Cultures, *n* = 4 per group. Two-way ANOVA (*Post hoc* Bonferroni test): *p < 0.05 vs. respective non-treated Control; ^††^*p* < 0.01 vs. 2 DIV cells.

To determine whether 2 DIV and 16 DIV microglia, despite the downregulation of miR-155 and miR-124 expression, were still able to mount an inflammatory response upon Aβ insult, we assessed common biomarkers of microglia activation. We started by evaluating the gene expression of the first line cytokines TNF-α, IL-1β and IL-6. We obtained a clear upregulation of all these pro-inflammatory cytokines in the young cultured 2 DIV cells (Figure 8A). The 16 DIV cells showed a 10-fold reduction in the mRNA expression of TNF-α, IL-1β and IL-6, as compared with the 2 DIV control cells (*p* < 0.01). These cells, although less markedly than the 2 DIV cells, reacted to Aβ exposure by significantly increasing TNF-α and IL-1β gene expression, but not that of IL-6. Since we and others previously showed that HMGB1 is released by LPS-treated N9 microglia (Cunha et al., 2016) and promote the synthesis of pro-IL-1β and pro-IL-18 (Jiang et al., 2012), as well as the activation of NLRP3-inflammasome (Chi et al., 2015), we additionally explored these signaling pathways in our differentially aged microglia model treated with Aβ. In accordance with the previous results on cytokines, we observed a net elevation of HMGB1 and IL-18 gene expression (1.6- and 2.1-fold, respectively, *p* < 0.05) in 2 DIV microglia exposed to Aβ. However, no changes on NLRP3 were observed. Again, significantly decreased levels were obtained for all biomarkers in 16 DIV microglia (*p* < 0.01), as compared with 2 DIV cells, which revealed to be almost unreactive to the Aβ stimulus (Figure 8B).

**Figure 9:**
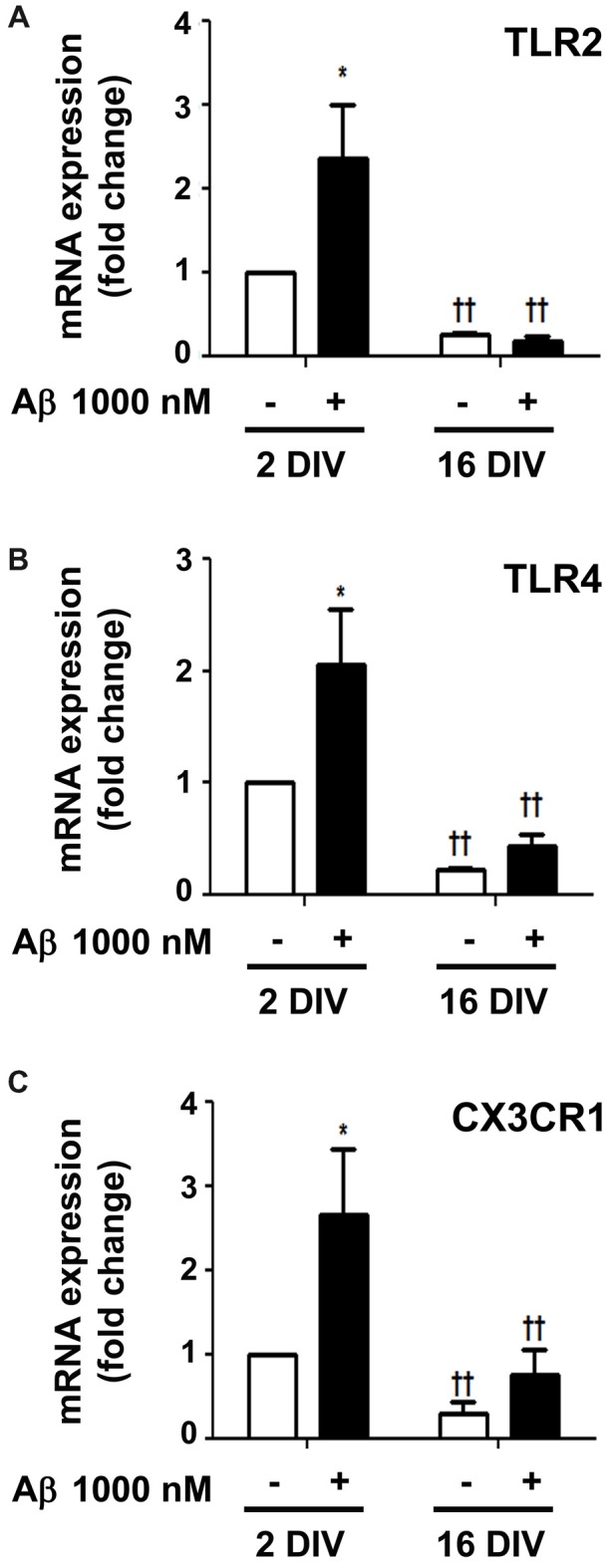
Amyloid-β (Aβ) peptide upregulates the expression of Toll-like receptor 2 (TLR2), TLR4 and fractalkine/CX3C chemokine receptor 1 (CX3CR1) in the reactive cultured microglia, but not in aged cells. Microglia that were kept in culture for 2 and 16 days *in vitro* (DIV) were treated with 1000 nM Aβ for 24 h. Expression of TLR2 **(A)**, TLR4 **(B)** and CX3CR1 **(C)** was evaluated by Real-Time PCR. Results are expressed in graph bars as mean ± SEM. Cultures, *n* = 4 per group. Two-way ANOVA (*Post hoc* Bonferroni test): *p < 0.05 vs. respective non-treated Control; ^††^*p* < 0.01 vs. 2 DIV cells; **(A)** DIV × Aβ interaction *F*_(5.29)_, *p* < 0.05.

Surface Toll-like receptors (TLRs) are abundantly expressed in microglia and recruitment of TLR2 and TLR4 by HMGB1 and IL-1β was shown to amplify inflammation (Park et al., 2004; Facci et al., 2014). In agreement with previous results, Aβ enhanced the expression of both TLR2 and TLR4 in 2 DIV cells (2.4- and 2.0-fold, *p* < 0.05, respectively), but not in 16 DIV microglia (interaction between DIV and Aβ treatment for TLR2 *F*_(5.29)_, *p* < 0.05, Figures 9A,B), which again revealed an already suppressed basal expression of these receptors. The fractalkine/CX3C chemokine receptor 1 (CX3CR1) signaling pathway was also previously demonstrated to modulate microglial activation (Limatola and Ransohoff, 2014). As documented for TLR2 and TLR4, a similar profile was obtained for the CX3XR1 expression in 2 DIV (2.6-fold, *p* < 0.05) and 16 DIV aged microglia (Figure 9C). Data reinforce the ability of 2 DIV cells to develop an effective inflammatory response upon Aβ exposure, and confirm the low responsiveness of 16 DIV microglia in conformity with more unresponsive/dormant and senescent-like phenotype of these cells.

### Imbalance of M1 and M2 Phenotypes in Aβ-Treated 2 DIV and 16 DIV Microglia Suggests the Formation of Different Cell Subsets

**Figure 10:**
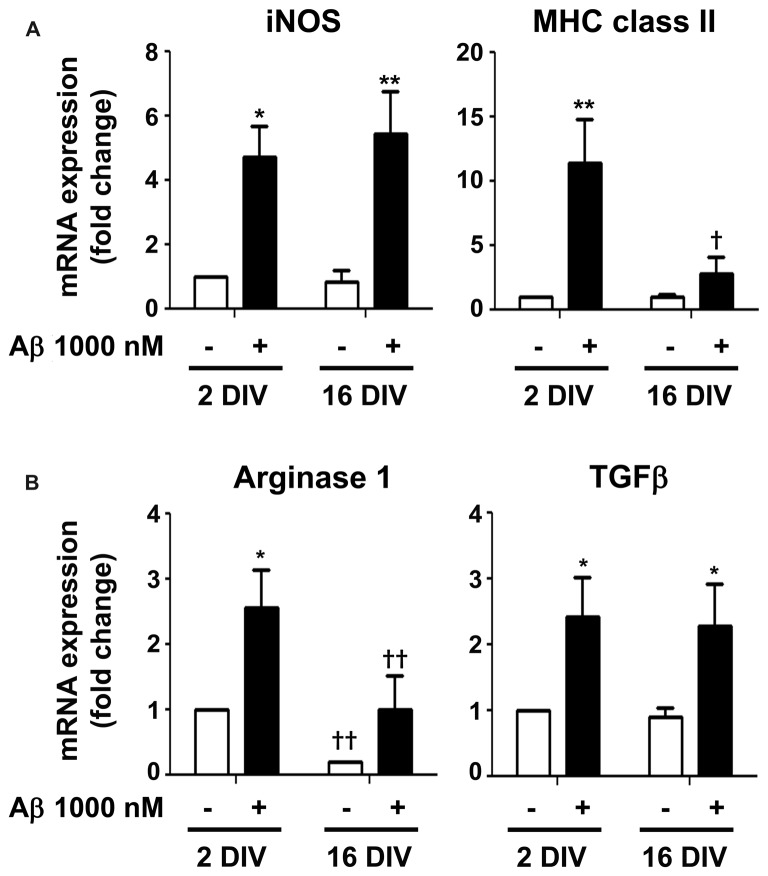
Mixed representation of M1/pro-inflammatory and M2/anti-inflammatory polarization markers in 2 and 16 days *in vitro* (DIV) microglia treated with amyloid-β (Aβ) peptide suggests the presence of different cell subsets. Microglia that were kept in culture for 2 and 16 DIV were treated with 1000 nM Aβ for 24 h. **(A)** Expression of M1/pro-inflammatory [e.g., inducible nitric oxide synthase (iNOS) and major histocompatibility (MHC) class II] and of **(B)** M2/anti-inflammatory [e.g., Arginase and transforming growth factor β (TGFβ)] was evaluated by Real-Time PCR. Results are expressed in graph bars as mean ± SEM. Cultures, *n* = 4 per group. Two-way ANOVA (*Post hoc* Bonferroni test): *p < 0.05 and **p < 0.01 vs. respective non-treated Control; ^†^*p* < 0.05 and ^††^*p* < 0.01 vs. 2 DIV cells; **(A)** MHC class II: DIV × Aβ interaction *F*_(4.53)_, *p* < 0.05.

From previous data, the elevation of IL-1β expression, mainly in 2 DIV microglia, indicates that the cell assumes preferentially the M1 phenotype upon Aβ exposure. However, the CX3CR1 increased expression also suggests microglia subclasses with the M2a polarization (Chhor et al., 2013). Therefore, we decided to further characterize the microglia phenotypes in 2 DIV and 16 DIV cells exposed to Aβ by evaluating additional M1 and M2 markers. For that we evaluated the gene expression of inducible nitric oxide synthase (iNOS) and of major histocompatibility (MHC) class II, which are considered M1/pro-inflammatory microglia markers, although MHC class II has also been attributed to M2b polarized macrophages (Roszer, 2015). As shown in Figure 10A, while iNOS was highly induced by Aβ in both young and aged microglia (4.7- and 5.4-fold for 2 and 16 DIV, *p* < 0.05 and *p* < 0.01, respectively), MHC class II was particularly high in young cultured cells (11.4-fold, *p* < 0.01, interaction between DIV and Aβ *F*_(4.53)_, *p* < 0.05), indicating a preferential M1 polarization in 2 DIV cells and a mixture of phenotypes in 16 DIV microglia. Then, we characterized M2/anti-inflammatory microglia markers, such as Arginase 1 (prevalent in M2a activation state) and transforming growth factor β (TGFβ) (suggested to be increased in the M2a/M2c/M2d subtypes (Roszer, 2015)). As observed in Figure 10B, Arginase 1, considered to be a repair/regenerative gene (Chhor et al., 2013), was only increased by Aβ in young cultured cells (2.6-fold, *p* < 0.05), with levels that, although slightly elevated upon Aβ, represented in the 16 DIV cells less than 40% (*p* < 0.01) of those in 2 DIV cells. In what concerns TGFβ expression, with neuroprotective and pro-survival properties (Dobolyi et al., 2012; Ryu et al., 2012), both young and aged Aβ-treated cells showed an increased expression (2.4- and 2.3-fold, respectively, *p* < 0.05). Overall, these results suggest that M1 and M2 subpopulations are present upon Aβ treatment. However, while 2 DIV cells mainly express M1 markers, a phenotypic dysregulation with overlapping of microglial M1 and M2 markers is present in aged microglia. These cells additionally showed a decreased ability to mount an adequate inflammatory response when stressed with Aβ.

### Proportion of CD11b and CD86 Positive Microglia Differs between 2 DIV and 16 DIV Cells after Incubation with Aβ

**Figure 11:**
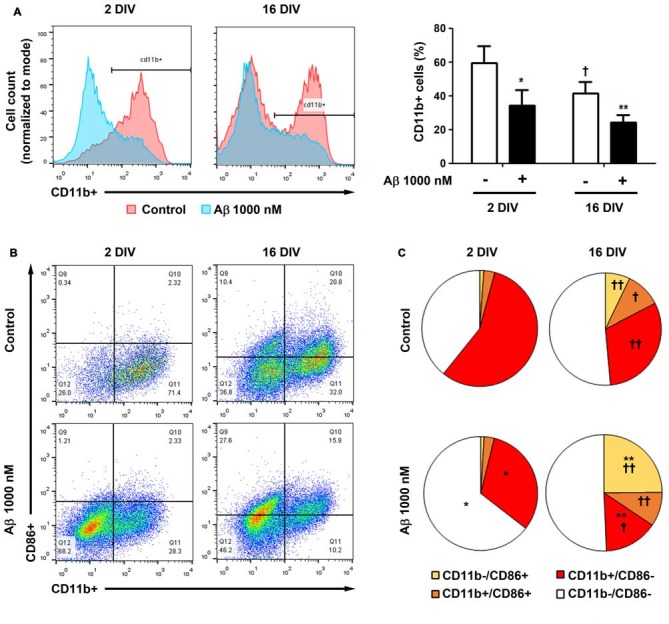
Cell aging and treatment with amyloid-β (Aβ) peptide decrease the population of microglia CD11b+ cells, while increase the number of CD86+ cells. Microglia that were kept in culture for 2 and 16 days *in vitro* (DIV)were treated with 1000 nM Aβ for 24 h. The population of CD11b+ and CD86+ cells was detected by flow cytometry. **(A)** Analysis of microglia expressing CD11b. Results are expressed in graph bars as mean ± SEM. Cultures, *n* = 4 per group. Two-way ANOVA (*Post hoc* Bonferroni test): *p < 0.05 and **p < 0.01 vs. respective non-treated Control; ^†^*p* < 0.05 vs. 2 DIV cells. **(B)** Representative flow cytogram of CD11b+ and CD86+ cells in reactive (2 DIV) and aged (16 DIV) cultured microglia. **(C)** Results are expressed in 2D pie graphs as mean. Cultures, *n* = 4 per group. Two-way ANOVA (*Post hoc* Bonferroni test): *p < 0.05 and **p < 0.01 vs. respective non-treated Control; ^†^*p* < 0.05 and ^††^*p* < 0.01 vs. 2 DIV cells.

To further understand whether the lower reactivity of 16 DIV microglia towards Aβ was associated with an increased expression of CD86, which was previously indicated to be age-related (Kohman et al., 2013), we evaluated changes in the proportion of the M1 markers CD11b+ (co-stimulatory ligand) and CD86+ (integrin αM) cells, in our microglia aged model of 2 DIV and 16 DIV after Aβ stimulus, by flow cytometry. As depicted in Figure 11A, the naïve aged microglia showed a decreased number of CD11b+ cells when compared to young/activated 2 DIV cells. In addition, these aged cells had a more elevated number of CD11b−/CD86− cells (~50%), together with elevated proportions of mixed CD11b−/CD86+ and CD11b+/CD86+ populations, than those showed by 2 DIV microglia, corroborating the aging-like profile status of 16 DIV cells (Figures 11B,C, Supplementary Table S3). When treated with Aβ both 2 DIV and 16 DIV cells showed a decreased population of CD11b+ cells. While 2 DIV microglia shifted from medium to high density in terms of CD11b−/CD86− cells, the number of CD11b−/CD86+ in 16 DIV microglia increased 4-fold (~25%) upon Aβ treatment and represented a 24-fold increase relatively to their 2 DIV counterparts (*p* < 0.01). No relevant changes were noticed when we assessed the expression of CD45 (data not shown). These results highlight that *in vitro* aging reduces CD11b+ microglia reactivity to Aβ. Increased CD86 signaling, namely in the presence of Aβ, further suggests the existence of M2b microglia with pro- and anti-inflammatory functions and a gain of function for co-stimulating other immune cells.

## Discussion

In the present manuscript we assessed whether *in vitro* aged microglia (Caldeira et al., 2014) and 2 DIV activated microglia differently reacted to Aβ stimulation, to better realize the complexity of microglia activation and cell dysfunctional processes in AD, as well as the relevance of immunosenescence to AD emergence. Actually, since AD pathophysiology is overlaid by the aging effects on the CNS, and microglia were shown to be dysfunctional in aging and AD (Mosher and Wyss-Coray, 2014; Cykowski et al., 2016), we aimed to recognize the relevance that microglia diverse phenotypes may have along the progression of the disease, and the role of subacute neuroinflammation in AD pathogenesis.

We observed that several neuroprotective functions, namely phagocytosis and migration abilities, as well as autophagy, were impaired by *in vitro* aging, contributing to Aβ deposition. Aged 16 DIV cells showed lower ability to mount an Aβ-induced inflammatory response with compromised expression of inflammation-related miRNAs and CD11b marker, but enhanced expression of the co-stimulatory CD86 molecule. In addition, our data pointed toward Aβ as a stressor-inducer molecule of microglia senescence. This is not without precedent since senescent astrocytes were shown to increase in human brain during aging and AD (Bhat et al., 2012), and dystrophic microglia was found in AD brain specimens (Streit et al., 2009). Findings support that aged microglia have compromised function and that Aβ reduces microglia ability to fully develop neuroprotective and inflammatory reaction against this noxious stimulus.

We have previously shown that impaired cell function by *in vitro* aging is not associated with loss of cell viability (Caldeira et al., 2014). Similarly, we did not observe age-dependent changes in cell death, either in the absence or in the presence of Aβ treatment. These results further validate our *in vitro* differently aged microglia model to evaluate perturbing effects by aging and Aβ. Changes in microglia morphology are associated with different functional states, where activation relates with larger somata, shorter processes and amoeboid morphology (Harry, 2013). Aged microglia also revealed to be smaller, less branched and less effective in mounting a normal response to injury. These cells with dystrophic appearance and less capacity to phagocytose and migrate, probably due to intracellular oxidative stress, were reported to be senescent (Streit et al., 2008), and to show increased ferritin immunoreactivity (Lopes et al., 2008). We previously demonstrated that cells acutely isolated and maintained for 2 DIV in culture behave as activated microglia, while if maintained in culture for 16 DIV exhibit a more bipolar shape and shorter large processes (Caldeira et al., 2014). Here, aged cells showed a thin and elongated shape with altered nuclei morphology. This type of cells, commonly called as rod cells, have been associated to chronically inflamed cerebral cortex (Hof and Mobbs, 2009) and acutely dementing processes (Graeber, 2010). As expected, when microglia were exposed to Aβ, in particular the 2 DIV cells, they acquired an amoeboid morphology, which is a morphometric characteristic of reactive microglia (Nakajima and Kohsaka, 2004). To note, however, that aged 16 DIV cells additionally showed distinct microglia morphological subclasses, as recently observed in the hippocampus of AD patients (Bachstetter et al., 2015). The elevated ferritin levels we observed in 2 DIV microglia reinforce activation by Aβ and suggest a putative defense mechanism against oxidative stress (Grundke-Iqbal et al., 1990; Yang et al., 2013). We also identified moderated accumulation of ferritin in aged cells, which is in accordance with dystrophic (senescent) microglia (Lopes et al., 2008) and may determine low resistance to ROS (Yang et al., 2013).

MMPs are important inflammatory components and their activation was shown to be implicated in AD pathogenesis (Wang et al., 2014). Besides their multiple roles in AD they are considered important for Aβ degradation (Miners et al., 2011). While 2 DIV cells secrete MMP9, but not MMP2, upon Aβ treatment, aged cells release both. MMP2 is considered the major protective gelatinase in AD and is overexpressed by astrocytes surrounding senile plaques, whereas MMP9 expression has a potential neurotoxic side and is described as a characteristic feature of AD (Wang et al., 2014). Indeed, activation of MMP9, but not of MMP2, was reported in serum and brain samples of patients with mild cognitive impairment and with AD (Lorenzl et al., 2008; Bruno et al., 2009). Increased release of MMP2 by 16 DIV cells may imply enhanced ability to cleave Aβ (Konnecke and Bechmann, 2013). Activation of MMP9 in both differentially aged cells may also disturb blood-brain barrier dynamic properties (Turner and Sharp, 2016), critically affecting brain Aβ homeostasis and its trans-endothelial transport and clearance (Provias and Jeynes, 2014).

Microglia migration is essential for many pathophysiological processes and a feature of the activated cell (Kettenmann et al., 2011). As in our previous study (Caldeira et al., 2014), aged microglia was unresponsive to ATP-induced chemotactic signals. In *ex vivo* retinal explants from aged mice (18–24 months of age), microglia process motility was reduced relatively to young adult animal cells (2–3 months of age; Damani et al., 2011). Interestingly, intranasally and intravenously administered microglia to mice migrate to brain in young and aged recipients, if derived from young, but not from aged donors (Leovsky et al., 2015). Such findings sustain microglia migration impairment with age. Again, young microglia, but not aged cells, were shown to migrate towards Aβ, following stimulation of ATP release by fibrillar and oligomeric Aβ_1–42_ species (Kim et al., 2012). Actually, microglial-mediated clearance of tissue debris was demonstrated to decay with aging (Neumann et al., 2009), to be compromised in older AD animal models (Njie et al., 2012), and to be associated with immunosenescence (Li, 2013). Functional impairment of microglia leading to phagocytic capacity decline was shown to coincide with Aβ deposition in a mice model of AD (Krabbe et al., 2013). In our *in vitro* aging microglia model the phagocytic ability of 2 DIV cells was decreased by Aβ, either in the number of beads per cell or maximum amount in each cell. Values obtained were at the same level of those presented by 16 DIV cells, whose phagocytic dysfunction was not modified by Aβ. Microglia phagocytosis is also related with the recognition of phosphatidylserine receptors following docking of the MFG-E8 molecule (Li, 2012). Neuroprotective effects of MFG-E8 against oligomeric Aβ toxicity were previously shown (Li et al., 2012), although phagocytosis of viable neurons may also occur, which is a disadvantage (Fricker et al., 2012). We were the first demonstrating MFG-E8 downregulation due to age and Aβ in primary cultures of microglia, though others have detected a reduced expression in AD patients (Boddaert et al., 2007).

AD risk is modulated by genetic factors that influence microglial activation. Most attractively, mutations in the Siglecs TREM2 and CD33 have been distinctly associated with the development of AD, and shown to act in opposing directions relatively to microglial activation and AD risk; alleles that inhibit TREM2 function increase AD risk, whereas alleles that inhibit CD33 function reduce such risk (Malik et al., 2013). TREM2 was indicated to support microgliosis (Zheng et al., 2017) and its deficiency to attenuate microglia phagocytic activity (Kawabori et al., 2015). Therefore, our results indicating an elevated expression of TREM2 in Aβ-treated 2 DIV microglia, but not in 16 DIV microglia, again point to a gain-of-function of the young cell relatively to the aged one. Furthermore, elevated expression of CD33 was observed in microglia treated with Aβ, mostly if aged. CD33 expression was reported to inhibit uptake and clearance of Aβ_1–42_ in microglial cell cultures and microglia immunoreactive for CD33 were shown to correlate with insoluble Aβ levels and plaque burden in the AD brain (Griciuc et al., 2013). In this sense, we observed that young cells expressing elevated TREM2 had enhanced internalization of Aβ, while aged cells with low TREM2 and upregulated CD33 showed reduced intracellular Aβ and increased number of extracellular deposits, further corroborating their inability to clear Aβ.

Upregulation of TLR2, TLR4 and CX3CR1 in 2 DIV cells upon treatment with Aβ, but not in 16 DIV cells, are in line with higher neuroprotection by young cells than by older ones. Increased expression of TLR2 and TLR4 was found in AD human brains and suggested to require stimulation by Aβ fibrils (Reed-Geaghan et al., 2009). Interestingly, TLR-signaling was shown to link the autophagy pathway to phagocytosis (Sanjuan et al., 2007) and to be involved in the clearance of Aβ deposits (Trudler et al., 2010). Role of CX3CR1 signaling in AD is still controversial. The ablation of CX3CR1 gene in a rodent AD model increased cytokine levels and Tau pathology, while also increased protofibrillar Aβ phagocytosis (Merino et al., 2016). However, CX3CR1 deficiency was associated with aberrant microglial activation and AD-related cognitive deficits (Cho et al., 2011). In another study, a protracted reduction of CX3CR1 expression in aged microglia was observed after lipopolysaccharide injection, together with incomplete resolution of inflammation and delayed recovery from sickness behavior (Wynne et al., 2010). Thus, the importance of CX3CR1 in AD is controversial and needs further clarification.

Altered TLRs/ligands interaction may derive from key signaling modulator miRNAs, particularly the trio miR-155, miR-21 ad miR-146a, during age-related changes of immune system functions (Olivieri et al., 2013a). Studies support the pivotal role of miRNAs in the regulation of microglial phenotype by promoting microglial quiescence (miR-124), or by driving microglial inflammatory and immune responses (miR-155 and miR-146a) (Ponomarev et al., 2013). While miR-124 was shown to be downregulated in hippocampal brain samples of AD patients from early to severe disease stages (Lukiw, 2007), miR-155 was reported to be overexpressed in circulating fluids and cells of AD individuals (Alexandrov et al., 2012; Guedes et al., 2016), as well as in 3xTg-AD mice brain (Guedes et al., 2014). However, in other works, miR-155 expression was found significantly reduced in old individuals (Noren Hooten et al., 2010). Our results indicate that both miR-124 and miR-155 are decreased in 16 DIV cells, and that their downregulation in 2 DIV microglia comes from Aβ interaction. Such reduction may have important consequences in AD progression since miR-155 and miR-124 were recognized as critical modulators of immunological responses and to possibly act as anti-inflammatory factors (Li et al., 2016; Qin et al., 2016).

TNF-α, IL-1β, IL-6, HMGB1 and IL-18, but not NLRP3, were increased in 2 DIV cells upon Aβ treatment, while only the first two were enhanced in the 16 DIV cells. A better understanding of the pro-inflammatory signaling pathways associated to AD is crucial to define their beneficial or harmful consequences, and if their targeting by NSAIDs is advantageous. Based on our data, we postulate that NSAIDs therapy should be envisaged as a stage-dependent disease strategy with potential benefits in early inflammatory states of AD disease, as suggested by others (Cole and Frautschy, 2010; Imbimbo et al., 2010; Wang J. et al., 2015). TNF-α and IL-1β increase is consensual in AD pathogenesis (Wang W. Y. et al., 2015) and was here observed in 2 DIV and 16 DIV microglia, which may then be considered as targets for selective tuning. We observed that Aβ was unable to stimulate the production of other inflammatory mediators in aged cells. Relationship between IL-6 concentration and aging is not clearly established, and although suggested to increase, implicated cell is not recognized and conflicting results have been published (Maggio et al., 2006). We have established that Aβ and LPS trigger the release of HMGB1 from microglia (Cunha et al., 2016; Falcão et al., 2017), and we observed its upregulation in the 2 DIV Aβ-treated microglia. HMGB1 is a nuclear protein acting as a co-factor for gene transcription. However, when in the extracellular fluid, it acts as an alarmin and a pro-inflammatory cytokine that signals through TLR2/TLR4 (Park et al., 2004), which were increased in the reactive microglia. HMGB1 is involved in AD pathology by inducing neurite degeneration (Fujita et al., 2016). Our results do not sustain NLRP3 activation, although clearly show upregulated IL-18, again more notoriously in 2 DIV than in 16 DIV cells. Although IL-18 has been indicated to be produced downstream of NLRP3 (Zaki et al., 2010), it was recently associated to NLRP1 inflammasome, as well (Murphy et al., 2016). In a recent article both inflammasome components were indicated to be activated in AD, but their direct association with microglia was not investigated (Saresella et al., 2016). Most interesting, increased expression of pro-IL-18 with defective NLRP3 activation was observed in dendritic cells from elderly mice during influenza infection, highlighting that IL-18 upregulation may occur in the absence of NLRP3 activation (Stout-Delgado et al., 2012).

The increase in pro-inflammatory cytokines, as well as in iNOS and MHC class II, indicates that Aβ triggers the polarization of microglia into the M1 phenotype (Wang W. Y. et al., 2015; Cunha et al., 2016), namely in the 2 DIV cultured microglia. Nevertheless, increased expression of TGFβ and Arginase 1, and in some cases also of MHC class II, suggests the presence of M2 subclasses in both differentially aged cells (Chhor et al., 2013; Roszer, 2015). Increased microglial iNOS and TGFβ signaling by aging and AD was observed in experimental models and patients (Dheen et al., 2005; Doyle et al., 2010; Mosher and Wyss-Coray, 2014; von Bernhardi et al., 2015). Actually, M1 and M2 phenotypes are the extreme subtypes of microglia polarization, and the existence of different heterogeneous activation states reflect the plastic nature of microglia (Bachstetter et al., 2015; Grabert et al., 2016). Heterogeneous populations of microglia in our model result from the co-existence of four separated CD11b−/CD86−, CD11b−/CD86+, CD11b+/CD86− and CD11b+/CD86+ subtypes in 2 DIV microglia, but more extensively in 16 DIV microglia. These distinct subsets may derive from differentiation dissimilarities, contributing to morphological and functional diversities. Major differences induced by Aβ included a decrease in the number of CD11b+ cells in 2 DIV cells and an increase in CD86+ cells in 16 DIV microglia. This aging-associated diversity is in line with previous studies showing that aged mice (22 months) have a greater proportion of CD86+ microglia in hippocampus than adult animals (4 months) (Kohman et al., 2013). CD86 was shown to have co-stimulatory effects on T cells activation (Tambuyzer et al., 2009) and Monsonego et al. (2003) demonstrated that Aβ-reactive T cell activation was CD86 microglia-dependent. Therefore, we hypothesize that aging and Aβ may potentiate interactions between microglia and infiltrating T cells, thus concurring for immune dysfunction. M2b polarized microglia with high MHC class II and CD86 expression, pro-/anti-inflammatory properties, and enhanced T cell recruitment capacity may be a subset of Aβ-treated 16 DIV cells. Loss of CD11b+ cells, containing the αMβ2 integrin receptor, in the Aβ-treated 2 DIV microglia can account to reduced phagocytic and migration abilities, since αMβ2 was reported to be implicated in phagocytosis, cell-mediated killing, chemotaxis and cellular activation (Cougoule et al., 2004; Chen et al., 2008).

In this study we used highly enriched primary cultures of microglia isolated from the cortex of mice pups, as described (Saura et al., 2003). A mild state of microglia activation was observed at 2 DIV cultures after the isolation procedure (Caldeira et al., 2014). Indeed, the calming state requires time in culture (Cristóvão et al., 2010). We used 2 DIV cells and treatment with 1000 nM Aβ to mimic activation of mild microglia-associated neuroinflammation, a risk factor for developing AD (Eikelenboom et al., 2012; Wang W. Y. et al., 2015). Considering that cellular senescence is interconnected with AD pathogenesis (Boccardi et al., 2015), and that microglial degeneration and loss of neuroprotection by the dystrophic/senescent microglia, rather than activated microglia, contributes to AD (Streit et al., 2009; Mosher and Wyss-Coray, 2014), we used *in vitro* 16 DIV aged microglia (Caldeira et al., 2014) and incubation with Aβ to also test this alternative hypothesis.

Data show that microglia activation by Aβ depends on the polarization state of the cell. If already activated, microglia react with increased migration and expression of all major inflammatory biomarkers (except NLRP3), but also showing dysfunctional consequences as low phagocytic ability, increased senescence-like behavior, decreased CD11b immunoreactivity and reduced inflammatory miR-155 and miR-124 expression. Changes are much less notorious in the mature/aged microglia that only respond by activation of MMP2 and MMP9, increased LC3-punta and CD86 immunostaining, together with elevated iNOS, TGFβ and TNF-α gene expression. Distribution of M1 and M2 polarized markers indicates that 2 DIV cells assume a predominant M1 phenotype in the presence of Aβ, while 16 DIV cells comprise diverse microglia subtypes that include M2 subclasses. Altogether, we hypothesize that diverse microglia polarized cells distinctly contribute to AD initiation and progression. However, given the complexity of AD and the involvement of multiple cell types, our results should be interpreted with caution and their translation to humans will require further studies.

New insights may be obtained with microglia isolated from human post-mortem brain tissue (Mizee et al., 2017), or derived from induced pluripotent stem cells generated from AD patients (Abud et al., 2017). Moreover, because neuron-astrocyte-microglia communication plays a crucial role in AD pathogenesis, and microglia activation triggers astrocyte neurotoxicity (Liddelow et al., 2017), additional studies using 3D culture models that allow cell-to-cell interplay and best recapitulate AD (Kim et al., 2015; Choi et al., 2016; Lee et al., 2016) should be used to corroborate or complement our findings.

## Author Contributions

DB conceived the project. AF and DB planned and designed the experiments. AF, ARV and ASF performed microglia cultures. CCaldeira performed the experiments. CCunha evaluated microRNA profiling. ARV assessed autophagy. AB and ES performed flow cytometry measurements and AF analyzed the results. CCaldeira, AF and DB interpreted experiments and wrote the manuscript. DB edited the final version. The manuscript has been read and approved by all named authors.

## Conflict of Interest Statement

The authors declare that the research was conducted in the absence of any commercial or financial relationships that could be construed as a potential conflict of interest.

## Supplementary Material

FIGURE S1 Reactive cultured microglia phagocytose higher amount of monomeric and dimeric species than the aged cells, which otherwise exhibit increased number of extracellular amyloid-β (Aβ) deposits. Microglia that were kept in culture for 2 and 16 days *in vitro* (DIV) were treated with 1000 nM Aβ for 24 h. **(A)** Representative images of Aβ immunoblots in cell lysates using the anti-Aβ clone W0–2 antibody. **(B)** Representative images of microglia immunostained for Iba1 (red) and Aβ (green) with nuclei staining (blue). Scale bar equals 50 μm.
