# Supplementary material for: Key Aging-Associated Alterations in Primary Microglia Response to Beta-Amyloid Stimulation
Source: Front Aging Neurosci. 2017 Aug 31;9:277. doi: 10.3389/fnagi.2017.00277 (PMC5583148; doi:10.3389/fnagi.2017.00277)
Supplement: Supplementary file 1 [file Table_1.docx]

**Supplementary Table 1 – List of primer sequences used for microRNA expression**

| **microRNA** | **Sequence (5’-3’)** |
| --- | --- |
| miR-124 | 5’-UAAGGCACGCGGUGAAUGCC-3’ |
| miR-146a | 5’-UGAGAACUGAAUUCCAUGGGUU-3’ |
| miR-155 | 5’-CTCAGAGAGGTGGAAGACCATGT-3’ |
| SNORD110 | Reference gene |
