# Supplementary material for: Key Aging-Associated Alterations in Primary Microglia Response to Beta-Amyloid Stimulation
Source: Front Aging Neurosci. 2017 Aug 31;9:277. doi: 10.3389/fnagi.2017.00277 (PMC5583148; doi:10.3389/fnagi.2017.00277)
Supplement: Supplementary file 2 [file Table_2.docx]

**Supplementary Table 2 – List of primer sequences used for gene expression**

| **Gene** | **Sense (5’-3’)** |  | **Antisense (5’-3’)** |
| --- | --- | --- | --- |
| MFG-E8 | TGACTTTGGACACACAGCGT |  | GTGTAGAACAACGGGAGGCT |
| TREM2 | AGCTACCCGCTACTGCAAAG |  | TCACTGCCAGGGGGTCTAAG |
| CD33 | CCAGCTCAATGTTACCCGGA |  | GCTTCCTGCCTCTTGATAGGA |
| TNF-α | TACTGAACTTCGGGGTGATTGGTCC |  | CAGCCTTGTCCCTTGAAGAGAACC |
| IL-1β | CAGGCTCCGAGATGAACAAC |  | GGTGGAGAGCTTTCAGCTCATA |
| IL-6 | CCGGAGAGGAGACTTCACAG |  | GGAAATTGGGGTAGGAAGGA |
| HMGB1 | CTCAGAGAGGTGGAAGACCATGT |  | GGGATGTAGGTTTTCATTTCTCTTTC |
| IL-18 | TGGTTCCATGCTTTCTGGACTCCT |  | TTCCTGGGCCAAGAGGAAGTG |
| NLRP3 | TGCTCTTCACTGCTATCAAGCCCT |  | ACAAGCCTTTGCTCCAGACCCTAT |
| TLR2 | TGCTTTCCTGCTGAAGATTT |  | TGTACCGCAACAGCTTCAGG |
| TLR4 | ACCTGGCTGGTTTACACGTC |  | GTGCCAGAGACATTGCAGAA |
| CX3CR1 | TGCTCTTCACGTTCGGTCTG |  | CTCAAGGCCAGGTTCAGGAG |
| iNOS | ACCCACATCTGGCAGAATGAG |  | AGCCATGACCTTTCGCATTAG |
| MHC class II | TGGGCACCATCTTCATCATTC |  | GGTCACCCAGCACACCACTT |
| Arginase | CTTGGCTTGCTTCGGAACTC |  | GGAGAAGGCGTTTGCTTAGTTC |
| TGF-β | CAGAGCTGCGCTTGCAGAG |  | GTCAGCAGCCGGTTACCAAG |
| Β-actin | GCTCCGGCATGTGCAA |  | AGGATCTTCATGAGGTAGT |
