# Supplementary material for: Key Aging-Associated Alterations in Primary Microglia Response to Beta-Amyloid Stimulation
Source: Front Aging Neurosci. 2017 Aug 31;9:277. doi: 10.3389/fnagi.2017.00277 (PMC5583148; doi:10.3389/fnagi.2017.00277)
Supplement: Supplementary file 3 [file Table_3.docx]

**Supplementary Table 3 – Flow cytometry analysis of the expression of CD11b and CD86 in microglia**

|  | **2 DIV** | |  | **16 DIV** | |
| --- | --- | --- | --- | --- | --- |
|  | **Control** | **Aβ** |  | **Control** | **Aβ** |
| **CD11b-/CD86+** | 1.15 (±0.72) | 0.96 (±0.15) |  | 7.16 (±3.06) | 24.87 (±6.45) *^††^ |
| **CD11b+/CD86+** | 3.08 (±0.71) | 2.88 (±0.21) |  | 10.26 (±4.17) ^†^ | 9.51 (±2.32) ^††^ |
| **CD11b+/CD86-** | 56.56 (±6.68) | 31.64 (±5.87) * |  | 31.12 (±3.48) ^††^ | 14.95 (±2.64) ** |
| **CD11b-/CD86-** | 39.21 (±9.54) | 64.52 (±9.03) * |  | 51.46 (±8.90) | 50.67 (±9.59) |

All results are means ± SEM from at least four independent experiments. Microglial cells were kept in culture for 2 days in vitro (DIV) and 16 DIV and treated with amyloid-β (Aβ) at 1000 nM for 24 h. The population of CD11+ and CD86+ expressing microglia was detected by flow cytometry using specific antibodies. Two-way ANOVA (Post-hoc Bonferroni test) *p<0.05, **p<0.01 *vs.* respective Control; ^†^p<0.05, ^††^p<0.01 *vs.* 2 DIV.
