# Supplementary figures and images for: Key Aging-Associated Alterations in Primary Microglia Response to Beta-Amyloid Stimulation
Source: Front Aging Neurosci. 2017 Aug 31;9:277. doi: 10.3389/fnagi.2017.00277 (PMC5583148; doi:10.3389/fnagi.2017.00277)

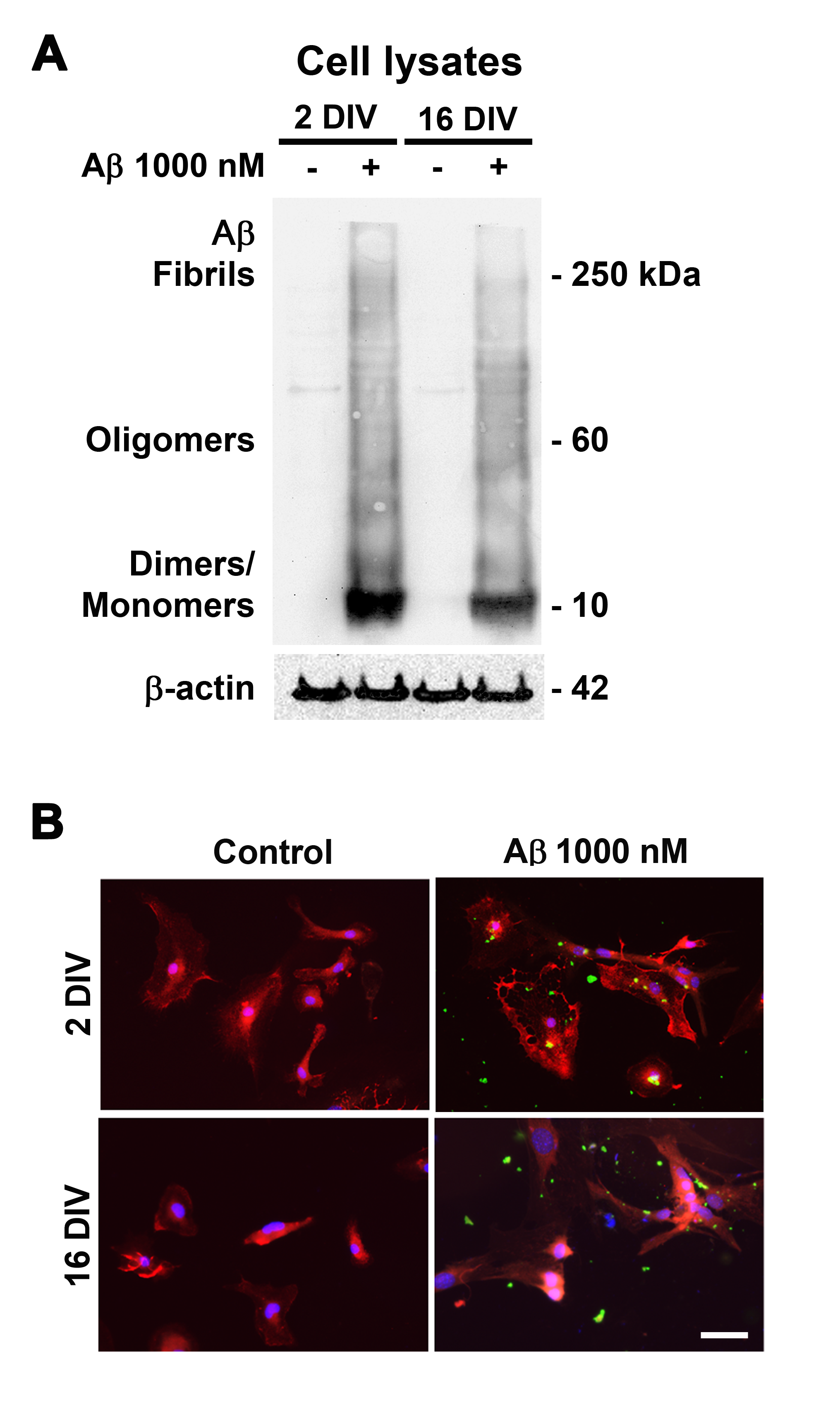

Supplement: FIGURE S1 — Reactive cultured microglia phagocytose higher amount of monomeric and dimeric species than the aged cells, which otherwise exhibit increased number of extracellular amyloid-β (Aβ) deposits. Microglia that were kept in culture for 2 and 16 days in vitro (DIV) were treated with 1000 nM Aβ for 24 h. (A) Representative images of Aβ immunoblots in cell lysates using the anti-Aβ clone W0–2 antibody. (B) Representative images of microglia immunostained for Iba1 (red) and Aβ (green) with nuclei staining (blue). Scale bar equals 50 μm. [file Image_1.tif]
